# The development and prevalidation of an *in vitro* mutagenicity assay based on MutaMouse primary hepatocytes, Part I: Isolation, structural, genetic, and biochemical characterization

**DOI:** 10.1002/em.22253

**Published:** 2018-12-27

**Authors:** Julie A. Cox, Edwin P. Zwart, Mirjam Luijten, Paul A. White

**Affiliations:** ^1^ Environmental Health Science and Research Bureau, Health Canada Ottawa Ontario Canada; ^2^ Department of Biology University of Ottawa Ontario Canada; ^3^ Centre for Health Protection National Institute for Public Health and the Environment (RIVM) Bilthoven The Netherlands

**Keywords:** transgenic rodent, liver, genetic toxicology, primary culture, metabolic enzymes

## Abstract

To develop an improved *in vitro* mammalian cell gene mutation assay, it is imperative to address the known deficiencies associated with existing assays. Primary hepatocytes isolated from the MutaMouse are ideal for an *in vitro* gene mutation assay due to their metabolic competence, their “normal” karyotype (i.e., neither transformed nor immortalized), and the presence of the MutaMouse transgene for rapid and reliable mutation scoring. The cells were extensively characterized to confirm their utility. Freshly isolated cells were found to have a hepatocyte‐like morphology, predominantly consisting of binucleated cells. These cells maintain hepatocyte‐specific markers for up to 3 days in culture. Analyses revealed a normal murine hepatocyte karyotype with a modal ploidy number of 4*n*. Fluorescence *in situ* hybridization analysis confirmed the presence of the lambda shuttle vector on chromosome 3. The doubling time was determined to be 22.5 ± 3.3 h. Gene expression and enzymatic activity of key Phase I and Phase II metabolic enzymes were maintained for at least 8 and 24 h in culture, respectively. Exposure to β‐naphthoflavone led to approximately 900‐ and 9‐fold increases in *Cyp1a1* and *Cyp1a2* gene expression, respectively, and approximately twofold induction in cytochrome P450 (CYP) 1A1/1A2 activity. Exposure to phenobarbital resulted in an approximately twofold increase in CYP 2B6 enzyme activity. Following this characterization, it is evident that MutaMouse primary hepatocytes have considerable promise for *in vitro* mutagenicity assessment. The performance of these cells in an *in vitro* gene mutation assay is assessed in Part II. Environ. Mol. Mutagen. 60:331–347, 2019. © 2018 The Authors. *Environmental and Molecular Mutagenesis* published by Wiley Periodicals, Inc. on behalf of Environmental Mutagen Society.

## INTRODUCTION

*In vitro* genetic toxicity tests are critical components of the toxicity assessment batteries typically employed for chemical safety evaluations and subsequent regulatory decisions (Kirkland et al., 2007). *In vitro* genetic toxicity assays currently used for regulatory purposes include both bacterial assays (e.g., the *Salmonella* reverse mutation test), as well as mammalian cell assays (e.g., the *hprt* and *xprt* gene mutation tests, the *in vitro* micronucleus assay, the mouse lymphoma assay (MLA), and the TK6 gene mutation assay). The current battery of *in vitro* genotoxicity assays has a lengthy history; indeed they have been prominent tools for protecting human health (Zeiger, 2010). In addition, attendant changes to the regulatory landscape, such as the Seventh Amendment to the European Union (EU) Cosmetics Directive, are stimulating increased reliance on *in vitro* tools that augment or even replace *in vivo* tests for routine chemical screening (European Commission, 2009; Adler et al., 2011; Tice et al., 2013). This shift away from *in vivo* models toward predictive *in vitro* tools, combined with the issues posed by specific mammalian *in vitro* tests, as discussed below, highlights the acute need to develop and adopt improved and/or alternative *in vitro* methods.

Although the aforementioned mammalian cell genotoxicity assays are highly sensitive, and have been well validated for routine use in regulatory assessments (i.e., Organization for Economic Cooperation and Development [OECD] Test Guidelines 476, 487, and 490) (OECD, 2016b; OECD, 2016c; OECD, 2016d), they present noteworthy drawbacks. First, none of the cell lines employed for these assays, such as the L5178Y, TK6, Chinese hamster ovary (CHO), Chinese hamster lung (CHL), and V79 cell lines, are metabolically competent, necessitating the use of exogenous activation mixtures containing, for example, Aroclor‐1254‐induced or phenobarbital/β‐naphthoflavone‐induced rodent liver S9 (Johnson et al., 1996; Cox et al., 2016). Unfortunately, the use of rodent liver S9 is problematic due to its cytotoxicity, the over‐representation of Phase I cytochromes P450 (CYPs), lack of Phase II enzyme activity, and poor penetration of exogenously formed metabolites into the cell (Glatt et al., 1981; Madle et al., 1986; Kirkland et al., 1989; Ku et al., 2007; Cox et al., 2016). Second, immortalized cells often used for genotoxicity assessment typically have aberrant and/or unstable karyotypes that include numerous deletions, duplications and translocations. Consequently, many commonly used cell lines, such as mouse lymphoma L5178Y *tk* ^*+/−*^ cells, show characteristics that are similar to oncogenically transformed tumor cells, including impaired p53 function (Storer et al., 1997). Genomic instability may also lead to genomic drift and subsequent differences in characteristics of the cell stocks used for routine genetic toxicity assessment (Lorge et al., 2016). It is anticipated that an *in vitro* assay that addresses these shortcomings could deliver more reliable and relevant results in comparison to existing *in vitro* genotoxicity assays.

Transgenic rodent (TGR) systems, such as the MutaMouse, have been shown to yield consistent and reliable results for detection of chemical mutagens and mutagenic carcinogens (Lambert et al., 2005; OECD, 2013). *In vitro* assays utilizing cells from TGR systems have previously been developed in an effort to complement the *in vivo* TGR assays. For example, a spontaneously immortalized cell line derived from the lung of the MutaMouse has been successfully employed in an *in vitro* gene mutation assay (White et al., 2003). This cell line, named FE1, exhibits significant benzo[*a*]pyrene (BaP)‐inducible *Cyp1a1*, *Cyp1a2*, and *Cyp1b1* gene expression; however, the addition of induced rat liver S9 is required to detect mutations induced by chemicals that are activated by other metabolic enzymes (White et al., 2003; Arlt et al., 2008; Berndt‐Weis et al., 2009). The FE1 *in vitro lacZ* gene mutation assay is currently undergoing validation according the multistep process advocated by the OECD (OECD, 2005).

More recently, mutagenicity assays using primary hepatocytes derived from both the MutaMouse and the pUR288 *lacZ* plasmid mouse have been developed (Chen et al., 2010; Zwart et al., 2012). Primary hepatocytes carry several advantages over immortalized cell lines, including endogenous metabolic competence and karyotypic stability. Indeed, the results of an *in vitro* gene mutation assay based on MutaMouse primary hepatocytes have shown concentration‐dependent increases in mutant frequency (MF) in response to BaP, 2‐amino‐1‐methyl‐6‐phenylimidazo[4,5‐*b*]pyridine (PhIP), and 3‐nitrobenzanthrone (3‐NBA), and a significant increase in MF following exposure to cigarette smoke condensate (CSC) (Chen et al., 2010). Both fresh and cryopreserved primary hepatocytes from the pUR288 *lacZ* plasmid mouse have been shown to proliferate in culture; moreover, they have BaP‐inducible CYP 1A1/1A2 activity as measured by ethoxyresorufin‐*O*‐deethylase (EROD), functional p53, and yielded results for 16 known mutagens and non‐mutagens that indicate excellent sensitivity and specificity (Zwart et al., 2012; Luijten et al., 2016). Collectively, these studies demonstrate the potential utility of primary hepatocytes from TGR systems as alternatives to existing mammalian cell *in vitro* mutagenicity tests.

Although the aforementioned study by Chen et al. (2010) showed that primary hepatocytes from the MutaMouse can be used to detect known mutagens, the cells and their various attributes have not been well‐characterized. The present study, which is Part I in a two‐part series, constitutes the next step in the development and establishment of an *in vitro* assay based on primary hepatocytes from the MutaMouse. More specifically, this work characterizes MutaMouse primary hepatocytes on the basis of their morphology, proliferative capacity, expression of markers indicative of cell type, karyotype, and metabolic capacity. Part II examines the performance of an *in vitro* gene mutation assay based on MutaMouse primary hepatocytes (i.e., the ability to effectively assess mutagenic hazard).

## MATERIALS AND METHODS

### Materials and Reagents

Dulbecco's modified Eagle's medium (DMEM), William's E medium, phosphate‐buffered saline (PBS), fetal bovine serum (FBS), epithelial growth factor (EGF), penicillin–streptomycin reagent, Hank's balanced salt solution (HBSS), trypan blue, colcemid, and Sytox® green nucleic acid stain were obtained from Life Technologies (Burlington, Ontario). Corning® Biocoat™ type I collagen‐coated culture dishes and coverslips were obtained from VWR International (Mississauga, Ontario). CIzyme™ collagenase HA and BP protease were obtained from VitaCyte LLP (Indianapolis, Indiana). American Type Culture Collection (ATCC) Eagle's minimum essential medium (EMEM), DMEM, and F‐12K medium were obtained from Cedarlane (Burlington, Ontario). VectaShield hardset mounting medium with 4′,6‐diamidino‐2‐phenylindole (DAPI) was obtained from Vector Laboratories (Burlington, Ontario). Dexamethasone, human insulin, dimethylsufoxide (DMSO), Percoll®, bovine serum albumin (BSA), resorufin ethyl ether, resorufin sodium salt, fluorescamine, ribonuclease (RNase) A, and IGEPAL CA‐630 were obtained from Sigma‐Aldrich Canada Co. (Oakville, Ontario). Bacteriophage lambda cl857 DNA was obtained from Roche Diagnostics (Laval, Quebec).

### Isolation and Culture of Primary Hepatocytes

The TGR MutaMouse (i.e., BALB/c × DBA2, mouse strain 40.6), carrying the bacteriophage lambda shuttle vector containing the bacterial *lacZ* target gene (Gossen et al., 1989), was bred and maintained locally under conditions approved by the Health Canada Ottawa Animal Care Committee. Hepatocytes were isolated from MutaMouse liver according to the two‐step collagenase technique proposed by Seglen (1976) with the addition of a Percoll® isodensity purification step (Kreamer et al., 1986). This study was restricted to female MutaMouse specimens that were not required for colony maintenance breeding purposes, thus primary hepatocytes were isolated from mice that ranged in age from 8 to 18 weeks. Primary hepatocytes were isolated from anesthetized mice following a retrograde perfusion using a blanching solution (10 mM 4‐(2‐hydroxyethyl)‐1‐piperazineethanesulfonic acid [HEPES], 1 mM ethylene glycol tetraacetic acid [EGTA], 100 U/mL penicillin–streptomycin in HBSS) and a collagenase‐containing solution (2,000 U/mL collagenase HA and 250 U/mL BP protease in DMEM), as previously described (Klaunig et al., 1981; Chen et al., 2010). The isolated cells were counted via hemocytometer using trypan blue exclusion. Successful perfusions yielded populations of hepatocytes that showed at least 80% viability. The cells were then plated onto collagen‐coated culture dishes using attachment medium (20 U/L human insulin, 4 × 10^−6^ mg/mL dexamethasone, 10% FBS, and 100 U/mL penicillin–streptomycin in DMEM), and incubated at 37°C and 5% CO_2_. Two hours (t = 2 h) following plating, the attachment medium was replaced with serum‐free medium (SFM; 10 mM HEPES, 2 mM L‐glutamine, 10 mM pyruvate, 0.35 mM l‐proline, 20 U/L human insulin, 4 × 10^−6^ mg/mL dexamethasone, 0.01 μg/mL EGF, and 100 U/mL penicillin–streptomycin in Williams medium E), and the plates incubated at 37°C and 5% CO_2_.

### Culture of Other Cell Lines

C2C12 mouse muscle myoblasts, RAW 264.7 mouse macrophages, A549 human lung carcinoma cells, and HepG2 human hepatocellular carcinoma cells were acquired from the ATCC through Cedarlane (Burlington, Ontario). C2C12 and RAW 264.7 cells were cultured in DMEM, A549 cells were cultured in F‐12K medium, and HepG2 cells were cultured in EMEM. All media were supplemented with 10% FBS and 100 U/mL penicillin–streptomycin. All incubations were carried out at 37°C and 5% CO_2_.

### Time‐Lapse Imaging

Time‐lapse videos of the primary hepatocytes in culture were captured using the JuLi Live Cell Movie Analyzer (NanoEnTek, Seoul, South Korea). Images were captured at 10× magnification at 10 min intervals, beginning 2 h following plating, for 120 h.

### Immunocytochemistry

Freshly isolated hepatocytes suspended in attachment medium, or cultured positive control cells (Supporting Information Table SI) suspended in their optimal medium, were plated onto collagen‐coated glass coverslips (hepatocytes) or sterilized uncoated glass coverslips (positive control cell lines) at 2.5 × 10^5^ cells/well in six‐well culture dishes, and incubated at 37°C and 5% CO_2_. After the hepatocytes had incubated for 2 h, the attachment medium was replaced with SFM, and the cells returned to the incubator for 24 or 72 h. Following 24 or 72 h incubation, the coverslips were fixed and permeabilized (if appropriate) in 4% paraformaldehyde and 0.1% Triton X‐100 in PBS for 15 min at room temperature. The coverslips were then incubated with 1% BSA in PBS with 2 mL/L Tween 20 (PBST) for 30 min to block nonspecific antibody binding. The coverslips were incubated with the primary antibody of interest in 1% BSA in PBST for 1 h at room temperature or overnight at 4°C. The coverslips were incubated with the secondary antibody for 1 h at room temperature in the dark. If necessary, this process was repeated for a second cell marker with a second set of primary and secondary antibodies. The antibodies used in these analyses are listed in Supporting Information Table SI. Antibodies against albumin and cytokeratin 18 were used to stain hepatocytes (Alpini et al., 1994; Wells et al., 1997). The presence of hepatic bile duct cells, fibroblasts, stellate cells, and Küpffer cells was determined using antibodies against cytokeratin 19, vimentin, desmin, and F4/80, respectively (Yokoi et al., 1984; Van Eyken et al., 1987; Kruglov et al., 2002; Li et al., 2014). The coverslips were mounted on glass slides using VectaShield hardset mounting medium containing DAPI and sealed with clear nail polish. Slides were imaged using a TCS SP8 confocal laser scanning microscope from Leica Microsystems (Concord, Ontario).

### Measurement of Nuclear Abundance

Relative nuclear abundance was measured to examine hepatocyte proliferation. Relative nuclear abundance was quantified by flow cytometry as described previously with some modifications (Nüsse et al., 1994; Avlasevich et al., 2006; Bryce et al., 2007). Briefly, cultured hepatocytes were lysed through the addition of Lysis Buffer I (0.584 mg/mL NaCl, 1 mg/mL sodium citrate, 0.5 μL/mL IGEPAL, 0.7 U/mL RNase A, and 0.5 μM SYTOX® green nucleic acid stain) directly to the plates following removal of SFM. Following incubation for 1 h in the dark at room temperature with gentle rocking, Lysis Buffer II (85.6 mg/mL sucrose, 15 mg/mL citric acid, and 0.5 μM SYTOX® green nucleic acid stain) was added to the plates, and the plates were incubated for an additional 30 min in the dark at room temperature with gentle rocking. To normalize nuclei counts, 150 μL of a suspension of 6 μm polystyrene microspheres was added to each sample of lysate. The microspheres are labeled with a fluorescent dye with excitation/emission maxima of 488/515 nm (Cell Sorting Set‐up Beads for Blue Lasers, Life Technologies, Burlington, Ontario). Each microsphere‐lysate sample was diluted 1:10 before flow cytometric analysis. Data were acquired using a BD Biosciences FACScalibur flow cytometer (BD Biosciences, Mississauga, Ontario) equipped with a 488 nm laser. Instrumentation settings and data acquisition were facilitated using CellQuest Pro software (BD Biosciences). Data analysis was performed using Flowing Software version 2.5.1 (Turku Centre for Biotechnology, Turku, Finland). SYTOX® green and bead fluorescence emission were captured in the FL1 channel (530/30 band‐pass filter). Events were scored as nuclei following the application of key criteria (i.e., within a side scatter (SSC) vs. forward scatter (FSC) region, within a region that excludes doublets, and within a FSC vs. FL1 region) (Supporting Information Fig. S1).

Nuclei counts were normalized to number of haploid genomes and presented relative to bead counts according to the following equation:

$$\frac{\left({\text{population}}_{2N}\times 2\right)+\left({\text{population}}_{4N}\times 4\right)+\left({\text{population}}_{8N}\times 8\right)}{{\text{population}}_{\text{beads}}},$$

whereas *population*_2*N*_ represents the number of events in the 2*n* population, *population*_*4N*_ represents the number of events in the 4*n* population, *population*_8*N*_ represents the number of events in the 8*n* population, *population*_beads_ represents the number of events in the bead population. These data were generated following the acquisition of at least 15,000 events, wherein events comprise both nuclei and beads.

The doubling time was calculated using the following equation:

$$\frac{\ln\left(2\right)}{a},$$

whereas *a* represents the slope of the linear portion of the relationship between the natural logarithm of the nuclei counts versus time. The doubling time was presented as the mean of five biological replicates (i.e., primary hepatocytes isolated from five different mice).

### Karyotype Analysis and Fluorescent *In Situ* Hybridization

Primary hepatocytes were seeded in 100 mm petri dishes at 1.2 × 10^6^ cells per dish. Two days post‐isolation at approximately 70% confluence, cultured hepatocytes were treated with 50 ng/mL colcemid in SFM for 1 h. Following colcemid treatment, the dishes were incubated with 1 mL of a 0.05% Trypsin–EDTA solution at 37°C and 5% CO_2_. Trypsinization was stopped after 5 min with the addition of attachment medium, and the cells were gently collected in 15 mL tubes and pelleted at 220*g* for 10 min. The cell pellet was gently resuspended in 75 mM KCl. After 15 min, 6–8 drops of fixative (3:1 methanol to acetic acid) were added to each tube and the tubes immediately centrifuged for 10 min at 1,000 rpm. The pellet was resuspended in 75 mM KCl and another 6–8 drops of cold fixative were added to each tube. The tubes were agitated to mix and then filled with cold fixative. Tubes were stored at −20°C overnight.

The G‐to‐FISH karyotype analysis was performed by The Centre for Applied Genomics (TCAG) at the Hospital for Sick Children (Toronto, Ontario). The fixed cells were mounted on slides and digested with pancreatin for 35 s before Giemsa staining. A probe for the transgene was prepared from bacteriophage lambda cI857 DNA (Roche Diagnostics, Laval, Quebec) and labeled with SpectrumOrange. A control probe corresponding to the 3Hv locus on mouse chromosome 3 was prepared and labeled with SpectrumGreen. The probe mixture consisted of one part lambda SpectrumOrange probe, one part mouse 3Hv locus SpectrumGreen probe, two parts mouse Cot‐1 DNA, and seven parts hybridization buffer (50% deionized formamide and 10% dextran sulfate in 2× saline‐sodium citrate buffer, pH 7). The probe mixture was denatured at 75°C for 5 min, and incubated at 37°C for 30 min to re‐anneal repetitive sequences to mouse Cot‐1 DNA. The slides were denatured at 65°C for 20 s before the denatured probe mixture was applied to the slides. The slides were hybridized overnight at 37°C in a lightproof, humidified oven. The chromosomes were counterstained with DAPI. As is the standard at TCAG, 20 metaphases were analyzed to allow for the detection of clonal chromosomal abnormalities with lower level mosaicism (Hook, 1977).

### Ethoxyresorufin‐*O*‐deethylase (EROD) Activity Assay

EROD is a measure of CYP 1A1 and 1A2 activity. Primary hepatocytes were suspended in 15 mL tubes at 1.2 × 10^6^ cells per 10 mL of attachment medium (i.e., for the 0 h timepoint) or seeded in 100 mm petri dishes at 1.2 × 10^6^ cells per dish (i.e., for the 2, 8, 24, and 48 h collection timepoints). The suspended hepatocytes were immediately centrifuged at 50*g* for 3 min, rinsed with PBS, frozen on dry ice and transferred to a −80°C freezer. Two hours post seeding, the medium for the plated hepatocytes was replaced with SFM or SFM containing 33 μM β‐naphthoflavone. At 2, 8, 24, and 48 h post seeding, dishes of cultured hepatocytes were rinsed with PBS, frozen on dry ice and transferred to a −80°C freezer. The EROD activity of primary MutaMouse hepatocytes was then measured using a modification of a method described previously (Kennedy and Jones, 1994; Kennedy et al., 1995). Resorufin was measured with excitation/emission wavelengths of 530/590 in nm and total protein was measured with excitation/emission wavelengths of 400/460 in nm using a SpectraMax Gemini EM Microplate Reader (Molecular Devices, San Jose, CA). Fluorescence values were converted to quantities of resorufin and total protein by comparison with simultaneously measured standard curves. EROD activity was measured for three biological replicates.

### Metabolite Analysis by LC–MS/MS

CYP 2B, CYP 3A, UDP‐glucuronosyltransferase (UGT), and sulfotransferase (SULT) activities were measured by liquid chromatography with tandem mass spectrometry (LC–MS/MS) quantification of testosterone and 7‐hydroxycoumarin metabolites. Briefly, primary hepatocytes were suspended in 15 mL tubes at 1.2 × 10^6^ cells per 10 mL of attachment medium or seeded in 100 mm petri dishes at 1.2 × 10^6^ cells per dish. The suspended hepatocytes were immediately treated with 100 μM testosterone or 200 μM 7‐hydroxycoumarin in SFM and incubated at 37°C with gentle shaking for 2 h. The suspended hepatocytes were then centrifuged at 50*g* for 3 min. Two hours post seeding, the medium for the plated hepatocytes was changed to plain SFM, SFM containing 33 μM β‐naphthoflavone, or SFM containing 100 μM phenobarbital. At 2, 8, 24, and 48 h post seeding, hepatocytes were treated with 100 μM testosterone or 200 μM 7‐hydroxycoumarin in SFM and incubated at 37°C and 5% CO_2_ for 2 h. Subsequently, the supernatant was removed and precipitated with two volumes of ice‐cold acetonitrile, shaken vigorously for 10 min, and centrifuged at 5,000*g* for 10 min to remove all particles. The particle‐free supernatant samples were analyzed for testosterone and 7‐hydroxycoumarin metabolites, specifically, 6β‐hydroxytestosterone, 16β‐hydroxytestosterone, 7‐hydroxycoumarin glucuronide, and 7‐hydroxycoumarin sulfate at Charles Rivers Laboratories (Cambridge, UK). Testosterone, 16β‐hydroxytestosterone, 6β‐hydroxytestosterone, and 7‐hydroxycoumarin were measured using a Xevo tandem quadrupole mass spectrometer (TQ‐MS) (Waters UK, Elstree, United Kingdom). 7‐Hydroxycoumarin sulfate and 7‐hydroxycoumarin glucuronide were measured using a Xevo TQ‐S (Waters UK, Elstree, United Kingdom). Instrument parameters, multiple reaction monitoring (MRM) parameters, and chromatographic conditions are provided in Supporting Information Tables SII–SV.

### Gene Expression

Primary hepatocytes were suspended in 15 mL tubes at 1.2 × 10^6^ cells per 10 mL of attachment medium or seeded in 100 mm petri dishes at 1.2 × 10^6^ cells per dish. RNA was isolated from cells 0, 2, 8, 24, and 48 h post‐isolation using Qiagen RNeasy kits (Toronto, Ontario), with three biological replicates, according to manufacturer's instructions. RNA quality was assessed by the Agilent RNA ScreenTape assay (Mississauga, Ontario) using the Agilent 2200 Tapestation System (Mississauga, Ontario) and all samples achieved RIN^e^ quality scores of at least 8.5. cDNA was synthesized using Qiagen RT^2^ First Strand kit (Toronto, Ontario) according to manufacturer's instructions. cDNA was prepared and applied to Qiagen Mouse Drug Metabolism RT^2^ profiler PCR arrays (catalog #PAMM‐002Z) (Toronto, Ontario) (Supporting Information Table SVI). The C_t_ values were determined using a BioRad CFX96 real‐time PCR thermal cycler (Mississauga, Ontario). A C_t_ cut‐off of 35 was applied.

### Statistical Analyses

Statistical analyses were performed using RStudio version 1.0.136 (RStudio, Boston, MA) software. Values are expressed as means ± standard error (SE). Comparisons between multiple conditions were performed with ANOVA, followed by Tukey's honest significance test. Real‐time qPCR data was normalized to the housekeeping gene, β‐2 microglobulin, and analyzed using the Livak method with significance calculated using the Students’ t‐test (Livak and Schmittgen, 2001). The significance of the slope of the nuclear proliferation data was assessed using least‐squares linear regression. The threshold for statistical significance was defined as *P* ≤ 0.05.

## RESULTS

**Figure 1:**
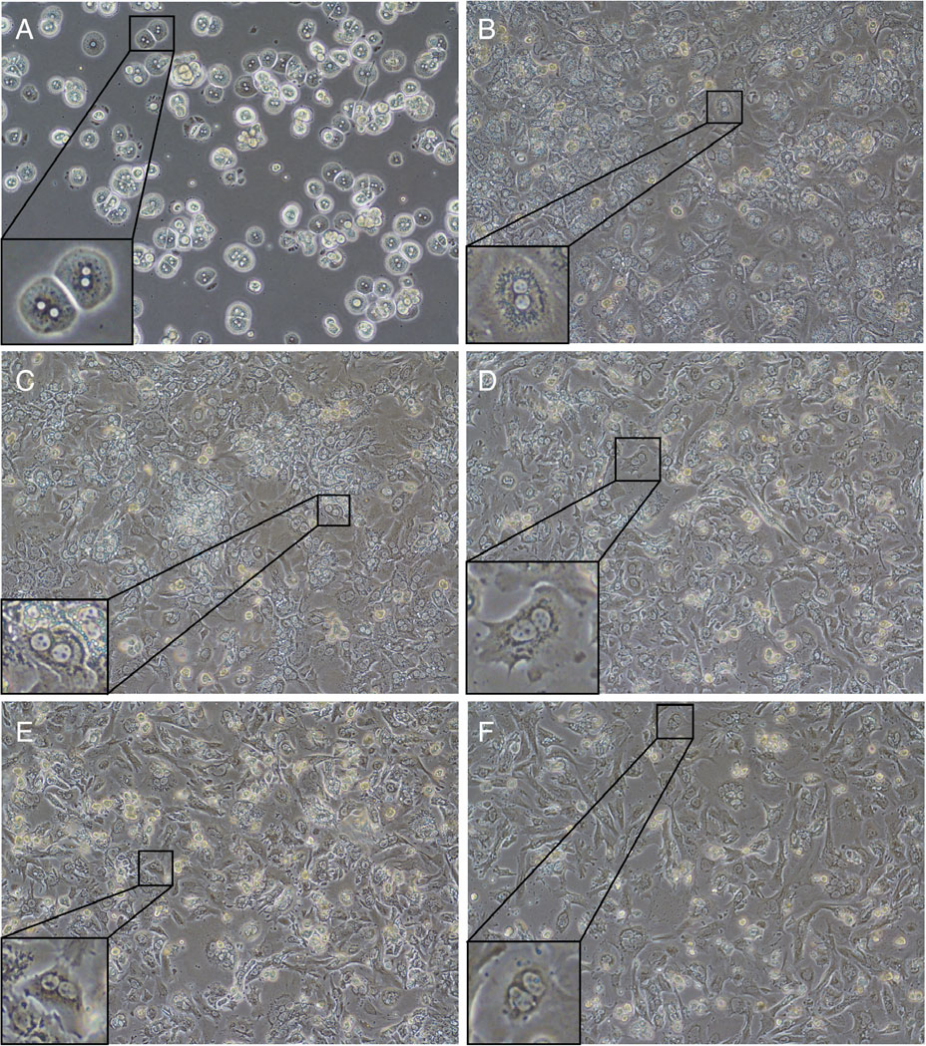
Time series of MutaMouse primary hepatocytes cultured on collagen‐coated petri dishes. Images acquired 2 (panel A), 24 (panel B), 48 (panel C), 72 (panel D), 96 (panel E), and 120 (panel F) h post‐isolation (100× magnification). Inset images display 300× magnified images of binucleated hepatocytes.

The appearance of the cells isolated from MutaMouse liver was examined by microscopy. The isolated cells are frequently binucleated (Fig. 1). Binucleated hepatocytes were visually enumerated in micrographs of five cultures and the proportion of binucleated cells was determined to be 78.1% ± 1.9% (data not shown). They present clear cytoplasms and cluster in small islands. Primary hepatocytes grown on collagen‐coated plates appear to maintain a cuboidal morphology for roughly the first 24 h, before developing a branched, spindle‐shaped appearance. This apparent de‐differentiation of the *in vitro* hepatocytes has been confirmed via time‐lapse imaging (Supporting Information Video).

**Figure 2:**
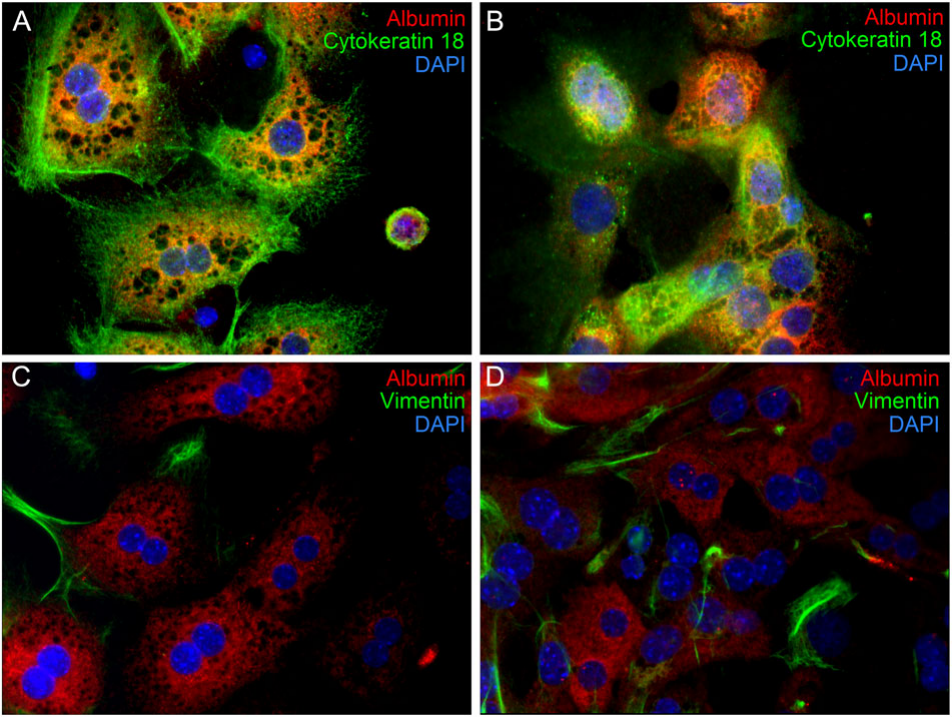
Representative immunofluorescent images of freshly isolated MutaMouse primary hepatocytes in culture for 24 (panels A and C) and 72 (panels B and D) h post‐isolation. Hepatocyte‐specific markers albumin (all panels) and cytokeratin 18 (panels A and B only) are shown in orange/red and green, respectively. The co‐expression of cytokeratin (green) and albumin (red) in panels A and B caused labeled albumin to appear orange; in contrast labeled albumin appears red in the lower panels. Marker of fibroblasts, vimentin (panels C and D only), is shown in green. Fixed cells were treated with primary antibodies, labeled with secondary antibodies, and counterstained with the nuclear stain DAPI (blue) (600× magnification).

Immunocytochemical analyses of cell‐specific markers permit the distinction between hepatic cell types. The results show that virtually all primary MutaMouse hepatocytes express albumin and cytokeratin 18, two hepatocyte‐specific markers, for at least 72 h in culture (Fig. 2A,B). The immunocytochemical analyses did not yield any evidence for the presence of hepatic bile duct cells, stellate cells, or Küpffer cells (data not shown). Vimentin staining, which was used to detect fibroblasts in the primary hepatocyte cultures, was sometimes observed as early as 1 day following hepatocyte isolation (Fig. 2, panel C), with staining increasing over time (Fig. 2, panel D). Roughly 10–20% of hepatocytes appear to express vimentin on Day 1, and roughly 50–70% of hepatocytes appear to express vimentin on Day 3.

**Figure 3:**
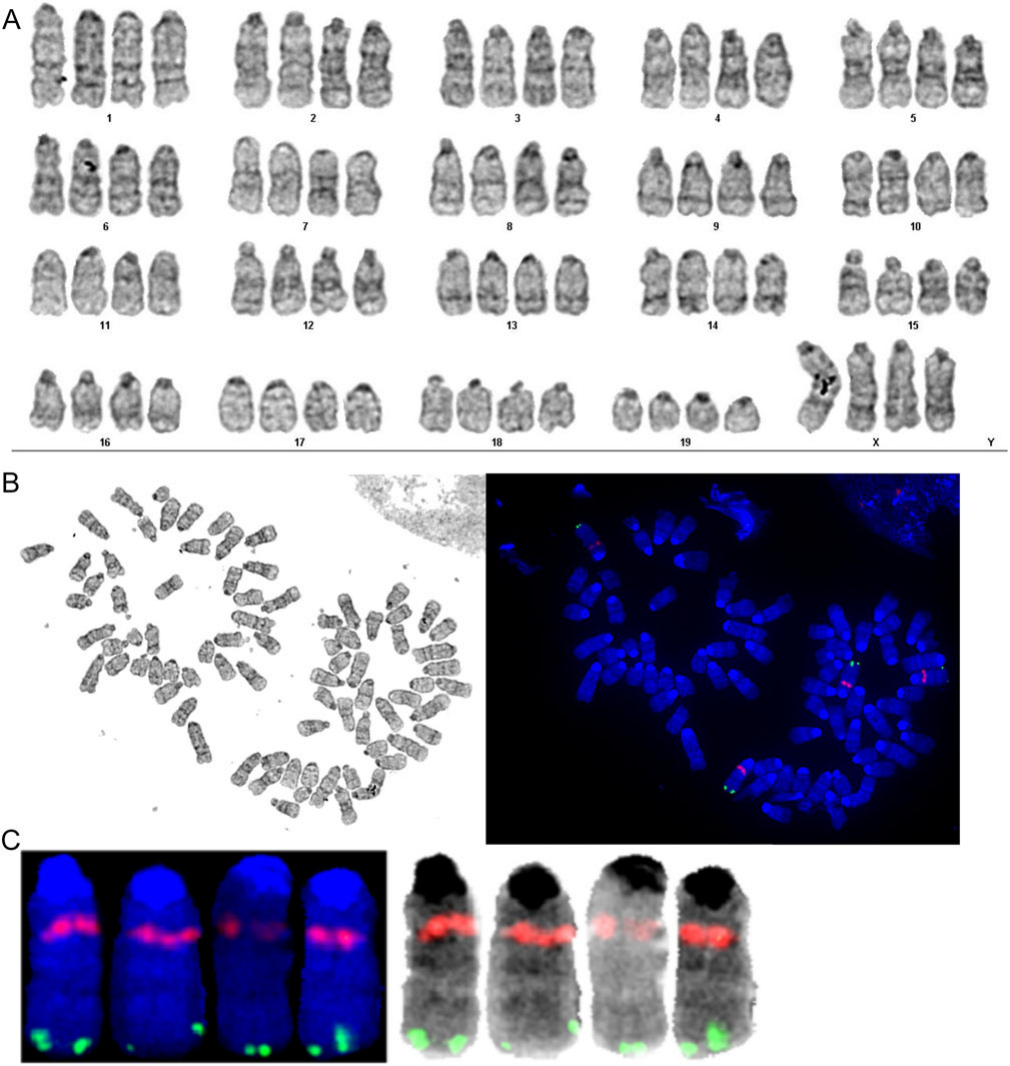
Representative karyotype and fluorescent *in situ* hybridization (FISH) results for cultured MutaMouse primary hepatocytes. Panel A shows a representative G‐banding karyotype of a 4*n* MutaMouse primary hepatocyte. Panel B shows the metaphase spread as seen in the original brightfield (left) and fluorescent (right) micrographs. Panel C shows representative FISH result on chromosome 3 as seen in the fluorescent micrograph (left), and with reverse‐DAPI banding (right), indicating λGT10 DNA (pink/orange) and the control H4 locus (green). The chromosomes were stained with DAPI, λGT10 DNA was labeled with SpectrumOrange, and a positive control probe consisting of mouse DNA from the chromosome 3 H4 locus was labeled with SpectrumGreen.

The karyotype of the cultured primary MutaMouse hepatocytes was assessed using a G‐to‐FISH analysis. The karyotype analysis revealed a modal chromosomal number of 80 (Fig. 3 and Table 1). Out of the 20 metaphases analyzed, 1 is 2*n*, 16 are 4*n*, 1 is 5*n*, and 2 are 8*n* (Table 1). Aneuploidy was evident in many of the metaphases analyzed. However, it should be noted that some instances of perceived aneuploidy may have been due to the technical artifact of metaphases overspreading. Five of the metaphases analyzed were found to have chromosomal aberrations, mainly chromosomal breakages, including terminal deletions. Fluorescence *in situ* hybridization (FISH) using bacteriophage λGT10 DNA labeled with SpectrumOrange, confirmed the presence of the *lacZ* transgene on chromosome 3 in all metaphases examined (Fig. 3 and Table 1).

**Table 1:** G‐to‐FISH Karyotype Summary

Number of cells	Chromosome count (ploidy)	FISH detection of bacteriophage λ DNA on chromosome 3 (number of signals detected)
1	160 (8*n*)^a^	Yes (8)
1	~153 (8*n*)^a^	Yes (8)
1	99 (5*n*)	Yes (6)
10	80 (4*n*)	Yes (4)
2	79 (4*n*)	Yes (4)
1	78 (4*n*)	Yes (4)
2	73 (4*n*)	Yes (3)
1	70 (4*n*)	Yes (3)
1	40 (2*n*)	Yes (2)

a Unclear whether these were octoploid cells or two proximate tetraploid nuclei.

**Figure 4:**
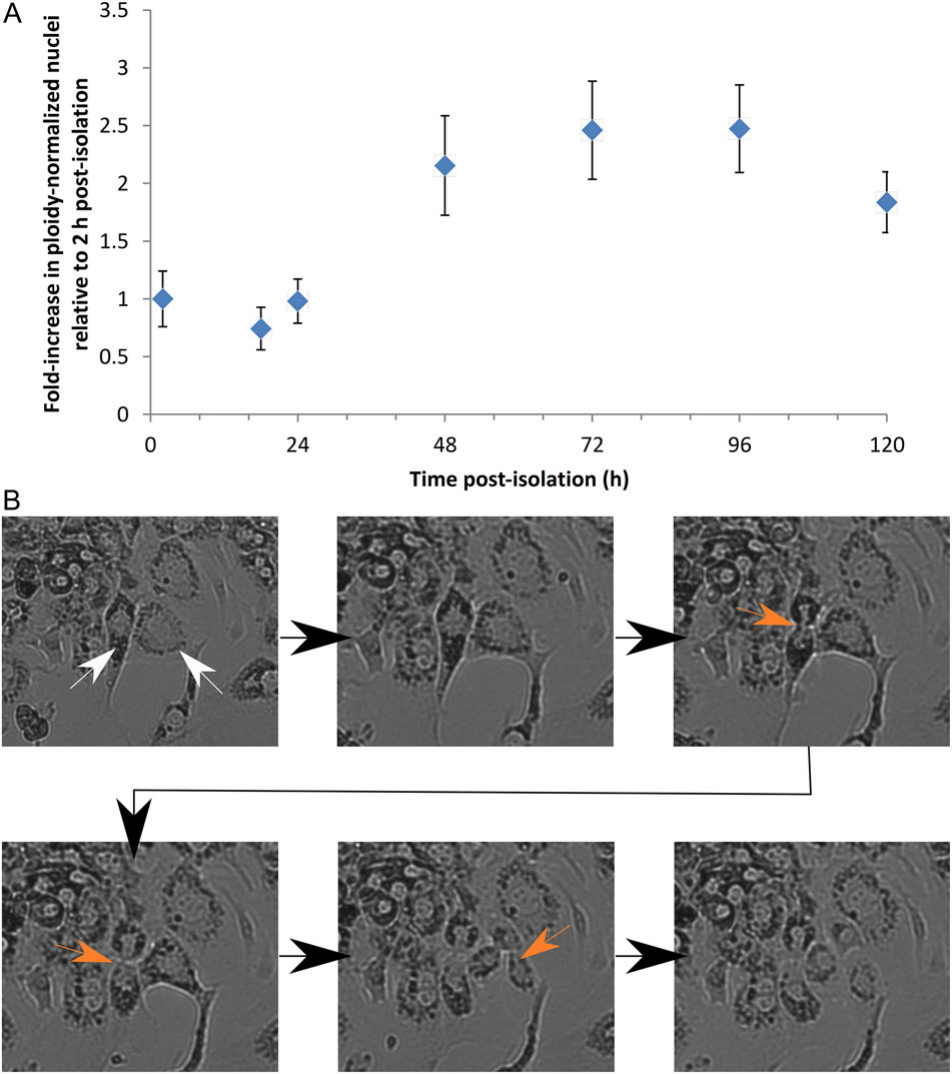
The proliferation of MutaMouse primary hepatocytes in culture. Panel A shows the temporal changes in the abundance of ploidy‐normalized MutaMouse hepatocyte nuclei. The mean fold‐increase in nuclei to bead ratio is presented (±SE; *n* = 5), relative to 2 h post‐isolation. Regression analysis revealed *r* ^*2*^ = 0.52, *P* < 0.005 for the linear portion of the relationship (i.e., 18–48 h), yielding a doubling time estimate of 22.5 ± 3.3 h. Sytox Green‐stained hepatocyte nuclei were mixed with a standardized volume of fluorescent beads, and temporal changes in abundance assessed using flow cytometry. Panel B is a representative sequence of both mononucleate and binucleate primary hepatocytes dividing in culture. The images (250× magnification), which were acquired 36 h post‐isolation at 10 min intervals, show two cells (white arrows) undergoing division (orange arrows). Time‐lapse video available as Supporting Information Video S1.

The proliferation of MutaMouse primary hepatocytes in culture was quantified using relative counts of nuclei measured by flow cytometry. Three discrete populations of nuclei were observed using this approach; they are presumed to represent the polyploid states observed via karyotypic analyses (i.e., 2*n*, 4*n*, and 8*n*) (Supporting Information Fig. S1). The three populations were normalized to their respective assumed ploidy number, pooled, and the nuclei/bead ratio was determined each day for five consecutive days following isolation (Fig. 4A). The calculated doubling time was 22.5 ± 3.3 h (*n* = 5). Hepatocyte proliferation was visually confirmed using time‐lapse microscopy (Fig. 4B and Supporting Information Video S1). Visual evaluation estimates the cell confluence to be ~30% at the start of culture, peaking at ~90% at 72 h, and falling to ~80% at 96 h, and 120 h.

**Figure 5:**
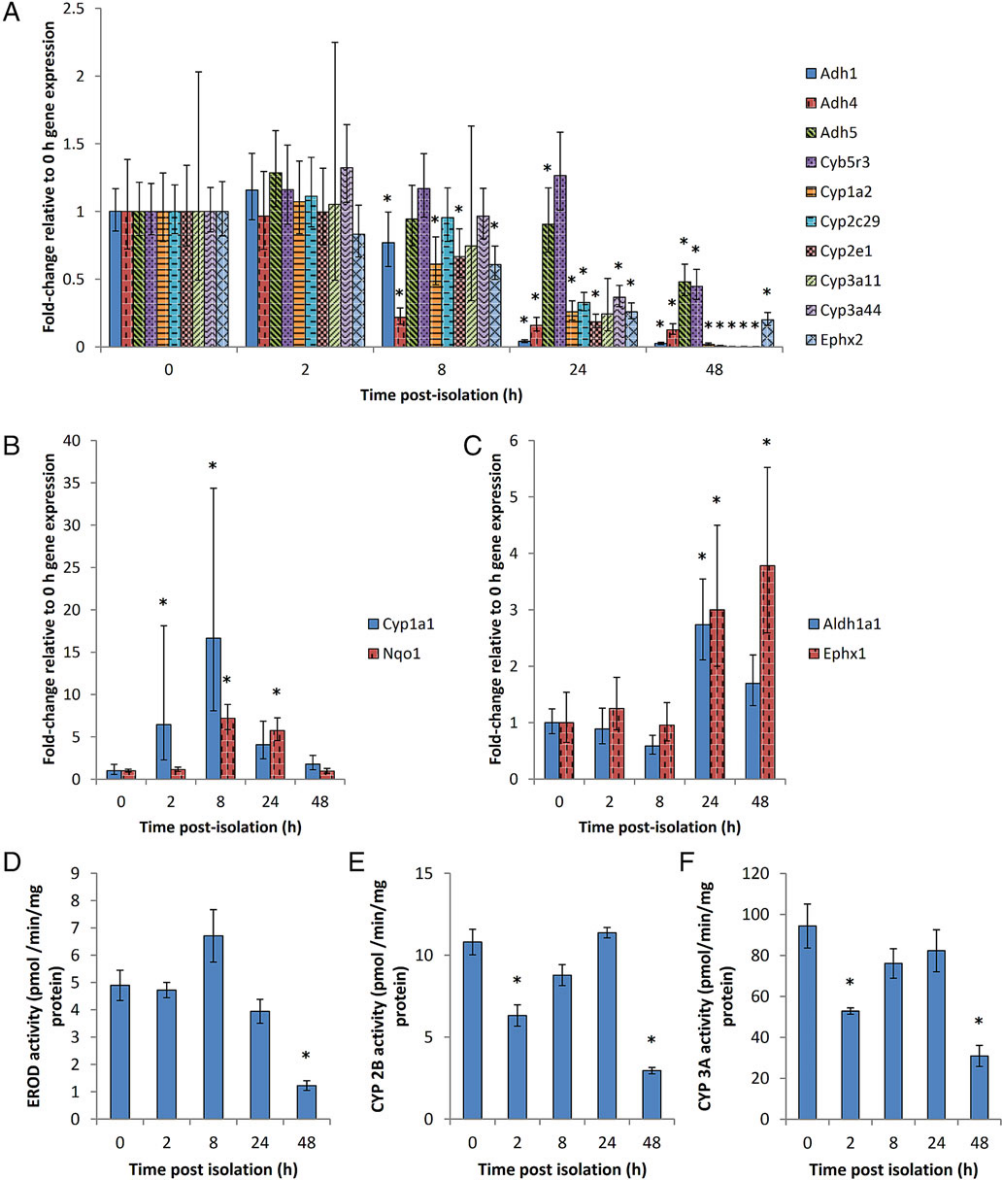
Temporal trends in gene expression (panels A–C) and enzyme activity (panels D–F) for key Phase I metabolic enzymes. Fold‐change gene expression changes of *Adh1*, *Adh4*, *Adh5*, *Cyb5r3*, *Cyp1a2*, *Cyp2c29*, *Cyp2e1*, *Cyp3a11*, *Cyp3a44*, and *Ephx2* (panel A), as well as *Cyp1a1* and *Nqo1* (panel B), and *Aldh1a1* and *Ephx1* (panel C), were quantified using a RT^2^ Profiler PCR array. The activity of CYP 1A1/1A2 was quantified by EROD (panel D). The activities of CYP 2B (panel E) and CYP 3A (panel F) were measured as testosterone 16β‐hydroxylation and testosterone 6β‐hydroxylation, respectively. *, significantly different from expression or activity at 0 h (*P* ≤ 0.05). Mean fold changes ± SE (*n* = 3) presented (i.e., relative to expression or activity 0 h post‐isolation). The genes encode the following metabolic enzymes: *Adh1*, alcohol dehydrogenase 1; *Adh4*, alcohol dehydrogenase 4; *Adh5*, alcohol dehydrogenase 5; *Cyb5r3*, cytochrome b_5_ reductase 3; *Cyp1a2*, CYP 1A2; *Cyp2c29*, CYP 2C29; *Cyp2e1*, CYP 2E1; *Cyp3a11*, CYP 3A11, *Cyp3a44*, CYP 3A44; *Ephx2*, epoxide hydrolase 2; *Cyp1a1*, CYP 1A1; *Nqo1*, NAD(P)H dehydrogenase, quinone 1; *Aldh1a1*, aldehyde dehydrogenase 1A1; *Ephx1*, epoxide hydrolase 1.

The Phase I metabolic enzyme capacity of MutaMouse primary hepatocytes was assessed using gene expression and enzyme activity analyses. The results show that the gene expression of many Phase I enzymes, including genes encoding a variety of CYPs, cytochrome b_5_ reductase, various alcohol dehydrogenases (ADHs), and epoxide hydrolase 2, as measured by real‐time qPCR, were stable until the 2 h post‐isolation time point; however, they begin to decline sharply 8 h post‐isolation (Fig. 5A and Supporting Information Table SVII). The gene expression of certain Phase I enzymes, such as *Cyp1a1*, encoding CYP 1A1, *Nqo1*, encoding NAD(P)H dehydrogenase, quinone 1 (NQO1), *Aldh1a1*, encoding aldehyde dehydrogenase (ALDH) 1A1, and *Ephx1*, encoding epoxide hydrolase 1, did not follow this trend. *Cyp1a1* exhibits 6.4‐ and 16.7‐fold increases in gene expression 2 and 8 h post‐isolation, respectively, followed by a return to the baseline expression level (Fig. 5B). Similarly, *Nqo1* showed 7.2‐ and 5.7‐fold increases in gene expression 8 and 24 h post‐isolation, respectively, followed by a return to the original expression level (Fig. 5B). *Aldh1a1* exhibited a 2.7‐fold increase in relative gene expression level 24 h post‐isolation before returning to the level observed in freshly isolated hepatocytes (Fig. 5C). The gene expression analysis of *Ephx1* reveals 3.0‐ and 3.8‐fold increases in relative expression 24 and 48 h post‐isolation, respectively (Fig. 5C). Interestingly, the catalytic activities of CYP 1A1/1A2, CYP 2B, and CYP 3A, measured by EROD activity, testosterone 16β‐hydroxylation, and testosterone 6β‐hydroxylation, respectively, remain fairly stable through the first 24 h post‐isolation, followed by significant reductions in enzyme activity at 48 h post‐isolation (Fig. 5D–F).

**Figure 6:**
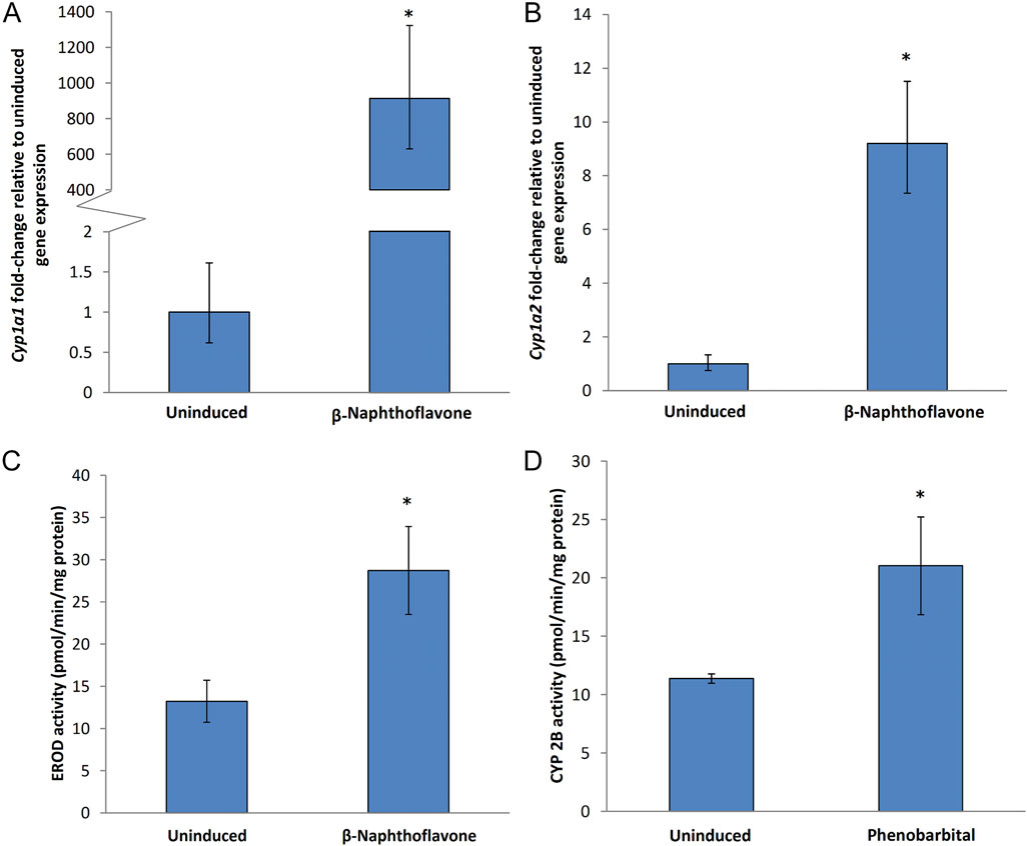
The expression and activities of key Phase I metabolic enzymes following 22 h induction with the Ah receptor agonist β‐naphthoflavone or the CAR agonist phenobarbital 24 h post‐isolation. β‐naphthoflavone‐induced changes in gene expression were observed for *Cyp1a1* (panel A) and *Cyp1a2* (panel B). The β‐naphthoflavone‐induced increase in the enzymatic activity of CYP 1A1/1A2 is shown in panel C. Phenobarbital‐induced increase in the enzymatic activity of CYP 2B is shown in panel D. Data presented as mean ± SE for triplicate samples, except panel C where *n* = 7. *, significantly different from control at *P* ≤ 0.05.

In addition to temporal changes in metabolic activity, the induction of gene expression and enzyme activity following exposure to aryl hydrocarbon receptor (AhR) and constitutive androstane receptor agonists (i.e., β‐naphthoflavone and phenobarbital) was investigated. Induced metabolic enzyme gene expression and activity were assessed 24 h post‐isolation. Exposure to β‐naphthoflavone elicited significantly enhanced expression of the genes encoding CYPs 1A1 and 1A2 approximately 900‐ and 9‐fold, respectively (Fig. 6A,B). CYP 1A1/1A2 enzyme activity (i.e., EROD activity) is also significantly increased (~twofold) following β‐naphthoflavone treatment (Fig. 6C). Exposure to phenobarbital yields a significant induction (~twofold) in CYP 2B enzyme activity, as measured by testosterone 16β‐hydroxylation (Fig. 6D).

**Figure 7:**
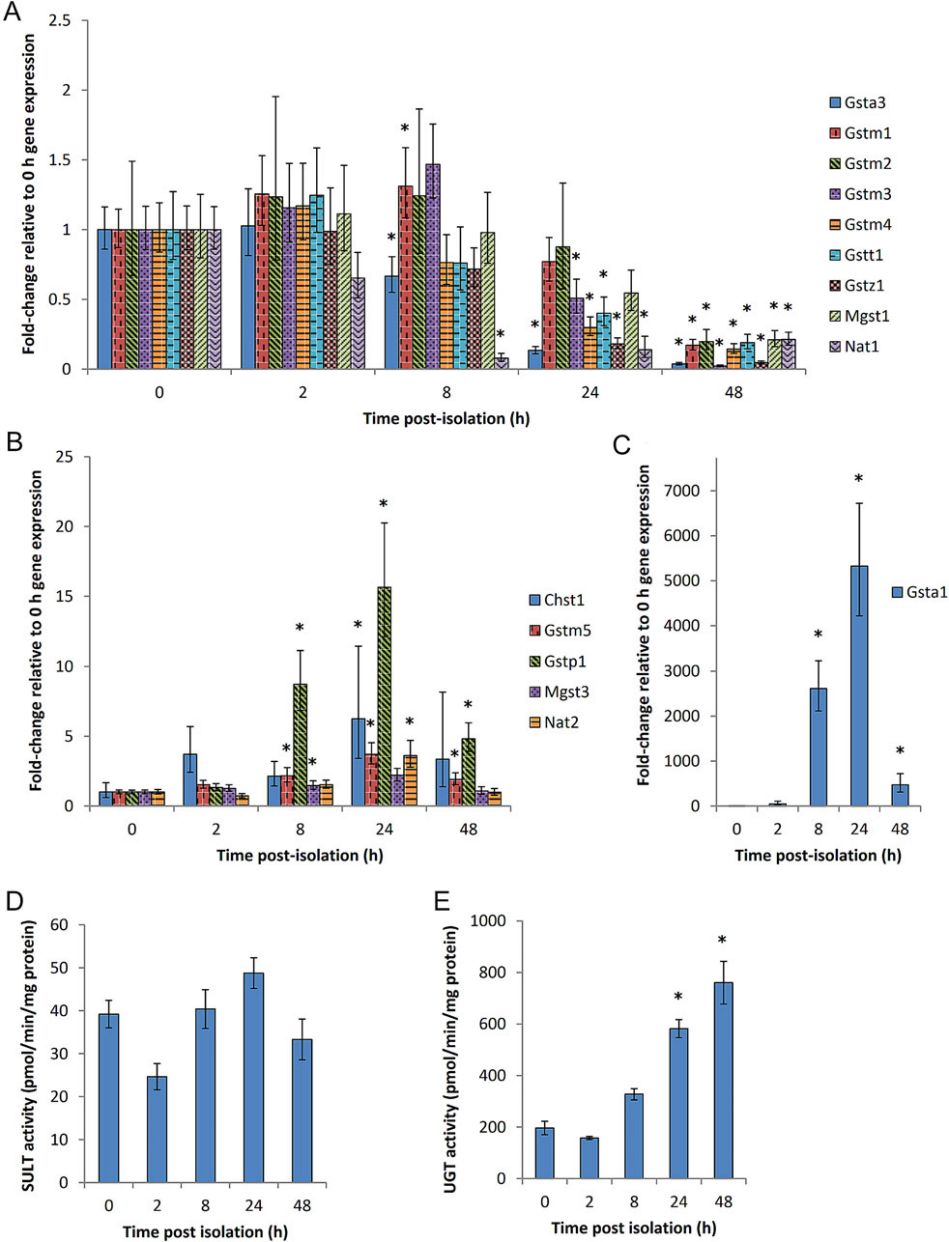
Gene expression (panels A, B, and C) and enzyme activity (panels D and E) of key Phase II metabolic enzymes. Fold‐changes in the gene expression of *Gsta3*, *Gstm1*, *Gstm2*, *Gstm3*, *Gstm4*, *Gstt1*, *Gstz1*, *Mgst1*, and *Nat1* (panel A), as well as *Chst1*, *Gstm5*, *Gstp1*, Mgst3*, and Nat2* (panel B), and *Gsta1* (panel C) were quantified using an RT^2^ Profiler PCR array. The activities of SULT (panel D) and UGT (panel E) were measured by 7‐hydroxycoumarin sulfation and 7‐hydroxycoumarin glucuronidation, respectively. *, significantly different from expression or activity at 0 h (*P* ≤ 0.05). Mean fold changes ± SE (n = 3) presented (i.e., relative to expression or activity 0 h post‐isolation). The genes encode the following metabolic enzymes: *Gsta3*, GST α1; *Gstm1*, GST μ1; *Gstm2*, GST μ2; *Gstm3*, GST μ3; *Gstm4*, GST μ5; *Gstt1*, GST θ1; *Gstz*, GST ζ1; *Mgst1*, microsomal GST 1; *Nat1*, NAT 1, *N*‐acetyl transferase 1; *Chst1*, carbohydrate SULT 1; *Gstm5*, GST μ5; *Gstp1*, GST π1; *Mgst3*, microsomal GST 3; *Nat2*, NAT 2; *Gsta1*, GST α1.

Gene expression and activity of Phase II metabolic enzymes were also assessed. Similar to the Phase I enzymes, the gene expression of several Phase II enzyme genes begins to decline 8 h post‐isolation (Fig. 7A and Supporting Information Table SVII). *Chst*, encoding carbohydrate SULT 1, *Gstm5*, encoding glutathione‐S‐transferase (GST) μ5, *Gstp5*, encoding glutathione‐S‐transferase (GST) π5, *Mgst3*, encoding microsomal GST 3, *Nat2*, encoding NAT 2, and *Gsta1*, encoding GST α1, are exceptions to this rule (Fig. 7B,C). *Chst* and *Nat2* shows 6.2‐ and 3.6‐fold increases in gene expression 24 h post‐isolation, respectively, followed by a return to original levels 48 h post‐isolation (Fig. 7B). Similarly, the gene expression of *Mgst3* shows a 1.5‐fold increase 8 h post‐isolation before returning to the baseline level (Fig. 7B). In contrast, *Gstm5* and *Gstp1* showed 2.2‐ and 8.7‐fold increases in gene expression 8 h post‐isolation, respectively, and do not return to original gene expression levels over the course of 48 h (Fig. 7B). Interestingly, *Gsta1* gene expression exhibits a several thousandfold increase over the matched 0 h control 8 and 24 h post‐isolation. At 48 h post‐isolation, the relative gene expression level of *Gsta1* decreases; however, it is still more than 400‐fold higher than the 0 h control (Fig. 7C). Contrary to the gene expression results, SULT enzyme activity, measured as 7‐hydroxycoumarin sulfation, shows no significant change over time relative to the enzymatic activity of freshly isolated cells (Fig. 7D) and UGT enzyme activity, measured as 7‐hydroxycoumarin glucuronidation, exhibits a gradual increase over time (Fig. 7E).

## DISCUSSION

The use of metabolically competent and karyotypically stable primary hepatocytes circumvents many of the disadvantages posed by currently employed *in vitro* mammalian cell genetic toxicity assays. Moreover, the use of primary hepatocytes from a TGR developed for *in vivo* scoring of induced somatic and germ cell mutations (i.e., OECD Test guideline 488) (OECD, 2013) would permit adoption of a well‐validated transgene mutation scoring system. Nevertheless, routine use of TGR primary hepatocytes is not without its challenges. Hepatocytes must be isolated, they must be capable of replicating in culture, and they must retain the metabolic capacity required for effective generation of DNA‐reactive metabolites. This study constitutes a thorough characterization of MutaMouse primary hepatocytes, including detailed information about their structural, biochemical, and karyotypic characteristics, and thus their potential to be used as the cornerstone of an *in vitro* mammalian cell gene mutation assay.

The protocol used to isolate MutaMouse primary hepatocytes results in robust cultures of essentially pure hepatocytes. The isolated cells are frequently binucleated, a known characteristic of mammalian hepatocytes (Fig. 1) (Gerlyng et al., 1993). MutaMouse primary hepatocytes in culture tend to closely associate with other hepatocytes in small clusters, which has been shown to enhance hepatocyte function (Dohda et al., 2003). Rodent hepatocytes *in vivo* are spherical (Klaunig et al., 1981; Arterburn et al., 1995). The isolated cells maintain a cuboidal, *in vivo*‐like appearance for the first 24 h in culture, followed by a temporal shift toward a flatter, spindle‐shaped, elongated morphology typically associated with fibroblasts (Fig. 1 and Supporting Information Video S1). This shift, known as de‐differentiation, has been well‐described *in vitro* (Elaut et al., 2006; Rowe et al., 2010). The de‐differentiation process involves a loss of phenotype, including hepatocyte‐specific functions, such as a gradual decline of metabolic activity, and results in the cultured hepatocytes more closely resembling generic, proliferative cells. A hallmark of the de‐differentiation process is the expression of vimentin (Godoy et al., 2009). Indeed, vimentin, a well‐known fibroblast marker, is increasingly expressed in cultured MutaMouse hepatocytes (e.g., 3 days post‐isolation, Fig. 3D). Vimentin staining in the culture is only observed in cells that are also expressing albumin, thus suggesting that these are hepatocytes undergoing de‐differentiation, rather than another cell type (e.g., fibroblasts). Moreover, the results show that the cultures are not contaminated by other liver cell types. Thus, the results obtained collectively indicate that the MutaMouse primary hepatocyte cultures are composed of healthy hepatocytes, and the cultures are not overtaken by a rapidly proliferating cell type (e.g., fibroblasts) over time in culture.

As noted, the karyotype analysis revealed extensive polyploidy and instances of aneuploidy, with attendant flow cytometric analyses confirming three distinct populations of nuclei (Table 1 and Supporting Information Fig. S1). Both the karyotype and flow cytometry results revealed that the 4*n* population is the most abundant. As mentioned, binucleation and polyploidization are common in hepatocytes (Guidotti et al., 2003; Duncan et al., 2010), and aneuploidy is also a known characteristic of mammalian hepatocytes both *in vivo* and *in vitro*. The latter has been postulated to be a mechanism for adaptation to stress; however, aneuploidy has been observed in normal, adult murine hepatocytes *in vivo* at a frequency of ~60% in the absence of any toxic insult (Duncan et al., 2010; Duncan et al., 2012). The karyotype analysis also confirmed the presence of the MutaMouse λGT10‐*lacZ* shuttle vector. Bacteriophage λGT10 DNA was identified using FISH, confirming the presence of the *lacZ* transgene on chromosome 3, as previously shown *in vivo* (Blakey et al., 1995). The presence of the *lacZ* transgene confirms that the isolated primary hepatocytes can be used to score induced mutations in much the same way as is currently done with the MutaMouse FE1 cell line (i.e., via the PGal positive selection assay) (Hoorn et al., 1993; White et al., 2003). Indeed, Part II documents the use of MutaMouse primary hepatocytes to reliably detect chemical mutagens, including those that require Phase I and/or Phase II metabolic capacity.

As cell division is required for mutagenesis, it is necessary to demonstrate that the isolated hepatocytes proliferate in culture. This is challenging as genomic and/or nuclear division in hepatocytes is often divorced from cytokinesis (Guidotti et al., 2003; Duncan et al., 2010). In other words, the hepatocyte genome replicates, but there is not necessarily an increase in cell number. The role of hepatocyte binucleation remains unclear, but it appears to be linked to polyploidization (Guidotti et al., 2003). Indeed, the hepatocytes observed herein are frequently multinucleated; moreover, flow cytometric analyses of isolated nuclei revealed three distinct populations of nuclei with increasing DNA content (Supporting Information Fig. S1). The karyotype analyses, discussed above, indicate that the recorded flow cytometric events are indeed indicative of 2*n*, 4*n*, and 8*n* nuclei. Cellular proliferation was observed using time‐lapse imaging, and the results showed unusual cytokinetic events that illustrate the complex kinetics of hepatocyte proliferation, such as a single hepatocyte generating three daughter cells (Fig. 4B and Supporting Information Video S1). Indeed, genomic and/or nuclear divisions without cytokinesis complicate measurement of growth rate, and temporal changes in the abundance of ploidy‐corrected nuclei were used to assess proliferation rate (Gerlyng et al., 1993; Guidotti et al., 2003). Analyses of this data (Fig. 4A) revealed a mean doubling time of 22.5 ± 3.3 h, similar to the doubling time of MutaMouse FE1 lung cells (White et al., 2003). The OECD test guideline for the *in vitro* mammalian chromosomal aberration test suggests a sampling time of at least 1.5 doubling times (OECD, 2016a). Given an adequate sampling time that allows for the variability around the MutaMouse primary hepatocyte doubling time (e.g., 72 h), this doubling time should permit fixation of transgene mutations. Importantly, the flow cytometry‐based method presented here, and utilized to measure primary hepatocyte proliferation, can easily be repurposed to assess chemically induced cytotoxicity using an adaptation of the “relative increase in cell count” (RICC) metric (i.e., relative increase in nuclear counts, or RINC) (Nüsse et al., 1994; Bryce et al., 2007; Avlasevich et al., 2006; OECD, 2016c).

Key metabolic enzymes that are involved in the activation of prototypical classes of bioactivated chemical mutagens have been detected in cultured MutaMouse primary hepatocytes (Table 2). Polycyclic aromatic hydrocarbons (PAHs) are activated to an electrophilic metabolite through a series of Phase I reactions, involving CYPs 1A1, 1A2, and 3A, as well as epoxide hydrolase (Bauer et al., 1995; Kim et al., 1998). Like PAHs, the metabolic activation of mutagenic mycotoxins, including aflatoxins, is mediated by CYPs, in particular, CYPs 1A2 and 3A (Gallagher et al., 1994). Nitrosamines, such as dimethylnitrosamine and diethylnitrosamine, are also activated by Phase I metabolism, specifically CYP 2E1 (Yamazaki et al., 1992; Chowdhury et al., 2012). Mutagenic phosphoramides, such as cyclophosphamide, require CYP 2B and CYP 3A for activation (Xie et al., 2003). Azoxyglycosides, such as plant‐derived cycasin, require the Phase I enzymes, CYP 2E1, ADH, and ALDH, for conversion to a mutagenic metabolite (McMahon et al., 1991; Sohn et al., 2001). The Phase I metabolic enzyme NQO1 has been postulated to have both azoreductase and nitroreductase activity and has been implicated in the activation of azo compounds and nitro‐PAHs (Huang et al., 1979; Møller and Wallin, 2000; Arlt et al., 2005). Aromatic amines (AAs), including 2‐acetylaminofluorene, and heterocyclic amines (HCAs), such as PhIP, generally require both Phase I (e.g., CYPs 1A1 and 1A2) and Phase II (e.g., SULT, NAT, and/or UGT) enzymatic reactions to generate DNA‐reactive nitrenium or carbenium ions (Heflich and Neft, 1994; Schut and Snyderwine, 1999; Cai et al., 2016). Although GSTs are well‐known for their detoxification and antioxidant functions, they have also been implicated in the mutagenic activation of halogenated hydrocarbons, such as 1,2‐dibromo‐3‐chloropropane (van Bladeren et al., 1980; Miller et al., 1986). The metabolic enzymes required in each of the examples above are present in MutaMouse primary hepatocytes, as measured by either enzyme gene expression or enzyme catalytic activity, thus illustrating their utility in an *in vitro* gene mutation assay.

**Table 2:** Catalytic Activity and Gene Expression of Metabolic Enzymes in MutaMouse Primary Hepatocytes. Selected Genes are Known to be Involved in the Activation of Prototypical Classes of Bioactivated Chemical Mutagens

Metabolic Enzyme	Chemical classes requiring enzyme for activation	Presence in MutaMouse primary hepatocytes	
		Activity	Expression
*Phase I*			
ADH	Azoxyglycoside	ND	Yes
ALDH	Azoxyglycoside	ND	Yes
CYP 1A1	PAH, AA, HCA	Yes	Yes
CYP 1A2	PAH, Mycotoxin, AA, HCA	Yes	Yes
CYP 2B^a^	Phosphoramide	Yes	ND
CYP 2E1	Nitrosamine, azoxyglycoside	ND	Yes
CYP 3A^b^	PAH, Mycotoxin, Phosphoramide	Yes	Yes
Epoxide hydrolase	PAH	NA	Yes
NQO1	Azo compound, Nitro‐PAH	ND	Yes
*Phase II*			
SULT	AA, HCA	Yes	Yes
NAT	AA, HCA	ND	Yes
UGT	HCA	Yes	ND
GST	Halogenated hydrocarbon	ND	Yes

Abbreviations: AA, aromatic amine; HCA, heterocyclic amine; ND, not determined; PAH, polycyclic aromatic hydrocarbon.

a Murine CYPs 2B9, 2B10, 2B13, 2B19, and 2B23 are closely related to human CYP 2B6 (Nelson et al., 2004).

b Murine CYPs 3A11, 3A13, 3A16, 3A25, 3A41, 3A44, and 3A57 are closely related to human CYP 3A4 (Nelson et al., 2004).

The temporal changes in enzyme gene expression profiles and activity levels were examined to further elucidate the utility of these cells in a gene mutation assay. This investigation demonstrates that the expression of many major metabolic enzyme genes in cultured MutaMouse primary hepatocytes is maintained for up to 8 h in culture, and the catalytic activity of major metabolic enzymes is maintained for at least 24 h (Fig. 5 A,D–F and Fig. 7A). The temporal discrepancy between expression and activity is likely due to differences between mRNA and protein turnover and stability (Yang et al., 2008). These results support findings by Mathijs et al. (2009) wherein the transcriptional changes in sandwich‐cultured murine hepatocytes were examined by microarray following 0, 42, and 90 h of cultivation. That study found that Phase I metabolic enzyme expression generally declined over time, and that Phase II gene expression either declined or showed no significant change (Mathijs et al., 2009). The maintenance of basal metabolic enzyme gene expression was generally prolonged in the Mathijs et al. study, relative to the study presented here, as has been observed in sandwich cultures versus monolayer cultures (Tuschl and Mueller, 2006). Collectively, the results presented herein indicate that MutaMouse primary hepatocytes are most metabolically active during the first 24 h of culture; therefore, the first 24 h of culture are the ideal timeframe for exposure to test chemicals that require metabolic bioactivation.

Although the activity and gene expression of many of the metabolic enzymes assessed in this study decline over time, there were some exceptions. Some enzymes, including *Cyp1a1*, *Nqo1*, *Aldh1a1*, *Chst1*, *Mgst3*, and *Nat2*, exhibit an initial increase in gene expression, followed by a return to the levels observed in freshly isolated hepatocytes (Fig. 5B,C and Fig. 7B). A similar trend is seen for *Ephx1*, *Gstm5*, and *Gstp1*, although the relative expression of these genes remains elevated 48 h post‐isolation (Fig. 5C and Fig. 7B). These expression patterns potentially indicate a delayed “recovery” of expression of these genes following an initial dampening of expression in freshly isolated hepatocytes, as has been seen for CYP1A1 in primary rat hepatocytes (Tuschl and Mueller, 2006). Interestingly, the relative gene expression of *Gsta1* increases dramatically to a 5,000 fold‐increase over freshly isolated hepatocytes 24 h post‐isolation. Upregulation of *Gsta1* has been linked to murine hepatocellular injury, and its increased expression over time in MutaMouse primary hepatocytes is likely due to trauma caused to the hepatic architecture during the cell isolation process (Liu et al., 2014). Mathijs et al. (2009) similarly noted upregulation for *Cyp1a1*, several GSTs, including *Gstm1* and *Gstm5*, and several NATs, including *Nat2*, over time. Similarly, UGT activity increases over time. UGT is known to have increased activity in response to membrane perturbants and preferentially metabolizes hydrophobic molecules (Bock, 1977), and UGT has also been implicated in the regulation of endogenous lipids, thus affecting proliferation and differentiation (Radominska‐Pandya et al., 1999; Dates et al., 2015). As hepatocyte isolation is an extremely disruptive process that unavoidably yields both healthy and damaged cells, harvest‐induced cellular stress may be leading to a sustained induction of UGT activity. Importantly, despite any potential cellular disruption during the isolation process, MutaMouse primary hepatocytes maintain metabolic competence for at least the first 24 h of culture and exhibit expression patterns typical of murine hepatocyte cultures.

Other than the use of induced rodent liver S9, there are currently few metabolically competent options for use in *in vitro* gene mutation assays. The HepaRG cell line is sometimes used in genetic toxicity assays where metabolic competence is desired due to its consistently high metabolic activity (Aninat et al., 2006; Lambert et al., 2009). Interestingly, comparisons of MutaMouse primary hepatocytes and HepaRG cells indicates that MutaMouse hepatocytes have ~4 to ~10‐fold higher activity for CYP1A1/1A2, SULT, and UGT, approximately equivalent activity for SULT, and one‐tenth the activity for UGT (Jossé et al., 2012; Kratochwil et al., 2017). Overall, the metabolic competence of MutaMouse primary hepatocytes is similar to, or exceeds, HepaRG cells. This comprehensive metabolic profile, coupled with the presence of the MutaMouse transgene, make these cells ideally suited for *in vitro* assessment of chemically induced mutations.

Primary human hepatocytes (PHHs) are the gold standard with respect to *in vitro* metabolic activity; thus, it useful to comparatively scrutinize the activity of the cells discussed herein with PHH cultures. EROD activity values for fresh PHHs are between 0.14 and 0.96 pmol/min/mg protein, 24 hours post seeding (Alexandre et al., 2002; Truisi et al., 2015); values for cryopreserved PHH cultures vary between 1.68 and 6.73 pmol/min/mg protein 96 hours post‐thawing (Roymans et al., 2005). PHH CYP 3A4 activity levels, measured by testosterone 6β‐hydroxylation, are between 26.6 and 67.4 pmol/min/mg protein 96 hours post‐thawing (Roymans et al., 2005); one study noted 55.0 pmol/min/mg protein for freshly cultured cells (Lübberstedt et al., 2011). Herzog et al. (2016) noted that UGT activity levels in freshly isolated PHH from two donors were 104.6 and 251.8 pmol/min/mg protein, respectively, as measured by hydroxycoumarin glucuronidation 48 h post seeding (Herzog et al., 2016). Thus, the values recorded for MutaMouse primary hepatocytes (Fig. 5D,F and Fig. 7E) are comparable to, or exceed, those recorded for PHHs.

Metabolic enzyme inducibility is indicative of the ability of primary hepatocytes to respond to xenobiotic insults. *Cyp1a1* and *Cyp1a2* gene expression is strongly induced following 22 h treatment with β‐naphthoflavone (i.e., ~900‐ and ~9‐fold, respectively). The increase in CYP 1A1/1A2 catalytic enzyme activity (i.e., ~twofold) is relatively modest, but the fold‐increase is similar to what has been observed in BaP‐induced pUR288 *lacZ* plasmid mouse hepatocytes (i.e., ~fivefold) (Zwart et al., 2012). The EROD activity observed in β‐naphthoflavone‐induced primary MutaMouse hepatocytes is ~100‐fold higher than that observed in β‐naphthoflavone‐induced rat primary hepatocytes (Lnenikova et al., 2018). In addition, the ~twofold induction in CYP1A1/1A2 and CYP2B activity observed in MutaMouse primary hepatocytes following exposures to β‐naphthoflavone and phenobarbital, respectively, is similarly observed in HepaRG cells (Wang et al., 2015). These results demonstrate that the inducibility of MutaMouse primary hepatocytes is similar to, or exceeds, that which is seen in other hepatocyte cultures.

By using a TGR mutation scoring system that is already internationally accepted and validated, and combining it with the metabolic competence and genetic stability of a normal primary hepatocyte, several of the problems plaguing current mammalian cell mutagenicity assays can be overcome. Indeed, utilization of hepatocytes from TGR models for *in vitro* mutagenicity assessment has already shown considerable promise (Chen et al., 2010;Zwart et al., 2012 ; Luijten et al., 2016). The results presented herein indicate that primary hepatocytes can readily be harvested from the MutaMouse; they are structurally and karyotypically normal and they proliferate in culture. The isolated hepatocytes are metabolically active for 24 h after isolation, and the observed activity is suitable for bioactivation of numerous known mutagens. Proliferation occurring after 24 h can permit the genetic damage to become fixed.

Routine use of primary hepatocytes from TGRs will require quality assurance criteria to ensure that the cells are functioning according to accepted standards. Cryopreservation of TGR primary hepatocytes has previously been described (Luijten et al., 2016), and could be employed to aid the distribution of MutaMouse primary hepatocytes. Although specification of precise quality assurance criteria will be necessary, it is not possible to precisely specify criteria at this time. Nevertheless, it is possible to provide some guidance with respect to the minimum acceptable level of metabolic activity. For example, based on levels recorded in this and other studies (Zwart et al., 2012; Luijten et al., 2016), it could be stated that TGR primary hepatocytes must have a baseline EROD activity level of at least 3 pmol/min/mg protein.

In conclusion, this work presents a thorough characterization of MutaMouse primary hepatocytes, in particular the cytological features that reflect their potential to be used for routine genetic toxicity assessments of new and legacy chemicals. Both this study and Part II focus on the use of MutaMouse primary hepatocytes in gene mutation assays; however, these cells could also be used *in vitro* for the assessment of chromosomal damage (e.g., micronucleus induction). The next step in the evaluation of their utility for routine chemical screening involves structured testing of selected mutagens and non‐mutagens, as recommended by the European Center for the Validation of Alternative Methods (EURL‐ECVAM) (Kirkland et al., 2008). Indeed, Part II presents mutagenicity assessments of nine known mutagens, two known non‐mutagens, and two compounds reported to elicit false positives *in vitro* and the results therein indicate high sensitivity and specificity. Part II also further discusses the criteria set out by the OECD Test Guideline program for the validation of novel toxicological test procedures and the additional criteria that this assay must meet before regulatory acceptance. It is anticipated that the attributes of MutaMouse primary hepatocytes, and their utility for chemical screening, will provide a foundation for their adoption as the cornerstone of a robust (i.e., sensitive and specific) *in vitro* mammalian cell mutagenicity assay that effectively complements existing *in vitro* tests (e.g., bacterial reverse mutation), and permits robust prioritization for follow‐up *in vivo* testing.

## AUTHOR CONTRIBUTIONS

Dr. White and Ms. Cox designed the study. Ms. Cox conducted the laboratory experiments, for which Drs. Zwart and Luijten had essential input. Dr. White and Ms. Cox analyzed the data. Ms. Cox prepared tables and draft figures. Ms. Cox prepared the draft manuscript with important intellectual input from Drs. White and Luijten. All authors approved the final manuscript.

## Supporting information


**Supplementary Table I**: Primary and secondary antibodies used for immunocytochemical analyses
**Supplementary Table II**: Settings for the electrospray ion sources used for the acquisition of testosterone, 16β‐hydroxytestosterone, 6β‐hydroxytestosterone, and 7‐hydroxycoumarin data with a Waters Xevo TQMS, and acquisition of 7‐hydroxycoumarin sulphate and 7‐hydroxycoumarin glucuronide data with a Waters TQS.
**Supplementary Table III**: Mass spectrometric detection parameters optimized via Multiple Reaction Monitoring (MRM) methods using Waters QuanOptimise. ES‐ electrospray ionization. + indicates positive ion.
**Supplementary Table IV**: UPLC gradient profile used for analysis of testosterone, 16β‐hydroxytestosterone, 6β‐hydroxytestosterone and 7‐hydroxycoumarin.
**Supplementary Table V**: UPLC gradient profile used for analysis of 7‐hydroxycoumarin sulphate and 7‐hydroxycoumarin glucuronide
**Supplementary Table VI**: Genes included in the Qiagen Mouse Drug Metabolism RT2 profiler PCR array. Genes shaded in green are housekeeping genes for normalization and genes shaded in grey are qPCR controls.
**Supplementary Table VII**: The fold‐changes in gene expression in MutaMouse primary hepatocytes over time for 84 murine metabolism. Expression measured using the Qiagen Mouse Drug Metabolism RT2 profiler PCR array. Red‐shaded cells indicate a significant fold‐increase, whereas green‐shaded cells indicate a significant fold‐decrease (*P* ≤ 0.05). Grey shading indicates lack of signal or a C_t_ value above the cut‐off of 35.
**Supplementary Figure 1**: Histogram and dot plots of primary MutaMouse hepatocyte nuclei illustrating the gates used to discriminate bead and nuclei populations from noise and spurious events. Events displayed and scored (panel A) were required to fall within the FL1 range (panel B), the light scatter region (panel C), the SSC vs FSC region (panel D), and the region that excludes doublets (panel E). The resulting FSC versus FL1 dot plot (panel A) displays a distinct bead population, as well as three populations representing 2n, 4n, and 8n nuclei.
**Supplementary Video 1**: Time‐lapse imaging of MutaMouse primary hepatocytes in culture. The imaging begins 2 hours post‐isolation and continues for 120 hours. Individual images were captured at 10 minute intervals at 10X magnification using bright‐field imaging.

**Appendix S1**: supplementary material for review

## References

[em22253-bib-0001] Adler S , Basketter D , Creton S , Pelkonen O , Van Benthem J , Zuang V , Andersen KE , Angers‐Loustau A , Aptula A , Bal‐Price A , et al. 2011 Alternative (non‐animal) methods for cosmetics testing: current status and future prospects‐2010. Arch Toxicol 85:367–485. 21533817 10.1007/s00204-011-0693-2

[em22253-bib-0002] Alexandre E , Viollon‐Abadie C , David P , Gandillet A , Coassolo P , Heyd B , Mantion G , Wolf P , Bachellier P , Jaeck D , et al. 2002 Cryopreservation of adult human hepatocytes obtained from resected liver biopsies. Cryobiology 44:103–113. 12151265 10.1016/s0011-2240(02)00011-1

[em22253-bib-0003] Alpini G , Phillips JO , Vroman B , Larusso NF . 1994 Recent advances in the isolation of liver cells. Hepatology 20:494–514. 8045510

[em22253-bib-0004] Aninat C , Piton A , Glaise D , Le Charpentier T , Langouët S , Morel F , Guguen‐Guillouzo C , Guillouzo A . 2006 Expression of cytochromes P450, conjugating enzymes and nuclear receptors in human hepatoma HepaRG cells. Drug Metab Dispos 34:75–83. 16204462 10.1124/dmd.105.006759

[em22253-bib-0005] Arlt VM , Gingerich J , Schmeiser HH , Phillips DH , Douglas GR , White PA . 2008 Genotoxicity of 3‐nitrobenzanthrone and 3‐aminobenzanthrone in Muta™Mouse and lung epithelial cells derived from Muta™Mouse. Mutagenesis 23:483–490. 18635558 10.1093/mutage/gen037

[em22253-bib-0006] Arlt VM , Stiborova M , Henderson CJ , Osborne MR , Bieler CA , Frei E , Martinek V , Sopko B , Wolf CR , Schmeiser HH , et al. 2005 Environmental pollutant and potent mutagen 3‐nitrobenzanthrone forms DNA adducts after reduction by NAD(P)H:quinone oxidoreductase and conjugation by acetyltransferases and sulfotransferases in human hepatic cytosols. Cancer Res 65:2644–2652. 15805261 10.1158/0008-5472.CAN-04-3544

[em22253-bib-0007] Arterburn LM , Zurlo J , Yager JD , Overton RM , Heifetz AH . 1995 A morphological study of differentiated hepatocytes in vitro. Hepatology 22:175–187. 7601410

[em22253-bib-0008] Avlasevich SL , Bryce SM , Cairns SE , Dertinger SD . 2006 In vitro micronucleus scoring by flow cytometry: differential staining of micronuclei versus apoptotic and necrotic chromatin enhances assay reliability. Environ Mol Mutagen 47:56–66. 16180205 10.1002/em.20170

[em22253-bib-0009] Bauer E , Guo Z , Ueng Y , Bell LC , Zeldin D , Guengerich FP . 1995 Oxidation of benzo[a]pyrene by recombinant human cytochrome P450 enzymes. Chem Res Toxicol 8:136–142. 7703357 10.1021/tx00043a018

[em22253-bib-0010] Berndt‐Weis ML , Kauri LM , Williams A , White P , Douglas G , Yauk C . 2009 Global transcriptional characterization of a mouse pulmonary epithelial cell line for use in genetic toxicology. Toxicol In Vitro 23:816–833. 19406224 10.1016/j.tiv.2009.04.008

[em22253-bib-0011] Blakey DH , Douglas GR , Huang KC , Winter NHJ . 1995 Cytogenetic mapping of gt10 lacZ sequences in the transgenic mouse strain 40.6 (MutaMouse). Mutagenesis 10:145–148. 7603331 10.1093/mutage/10.2.145

[em22253-bib-0012] Bock K . 1977 Dual role of glucuronyl‐ and sulfotransferases converting xenobiotics into reactive or biologically inactive and easily excretable compounds. Arch Toxicol 39:77–85. 414696 10.1007/BF00343277

[em22253-bib-0013] Bryce SM , Bemis JC , Avlasevich SL , Dertinger SD . 2007 In vitro micronucleus assay scored by flow cytometry provides a comprehensive evaluation of cytogenetic damage and cytotoxicity. Mutat Res 630:78–91. 17434794 10.1016/j.mrgentox.2007.03.002 PMC1950716

[em22253-bib-0014] Cai T , Yao L , Turesky RJ . 2016 Bioactivation of heterocyclic aromatic amines by UDP glucuronosyltransferases. Chem Res Toxicol 29:879–891. 27032077 10.1021/acs.chemrestox.6b00046 PMC4868641

[em22253-bib-0015] Chen G , Gingerich J , Soper L , Douglas GR , White PA . 2010 Induction of lacZ mutations in Muta™Mouse primary hepatocytes. Environ Mol Mutagen 51:330–337. 19953605 10.1002/em.20540 PMC2959491

[em22253-bib-0016] Chowdhury G , Calcutt MW , Nagy LD , Guengerich FP . 2012 Oxidation of methyl and ethyl nitrosamines by cytochrome P450 2E1 and 2B1. Biochemistry 51:9995–10007. 23186213 10.1021/bi301092c PMC3525961

[em22253-bib-0017] Cox JA , Fellows MD , Hashizume T , White PA . 2016 The utility of metabolic activation mixtures containing human hepatic post‐mitochondrial supernatant (S9) for in vitro genetic toxicity assessment. Mutagenesis 31:117–130. 26712374 10.1093/mutage/gev082

[em22253-bib-0018] Dates CR , Fahmi T , Pyrek SJ , Yao‐Borengasser A , Borowa‐Mazgaj B , Bratton SM , Kadlubar SA , Mackenzie PI , Haun RS , Radominska‐Pandya A . 2015 Human UDP‐glucuronosyltransferases: effects of altered expression in breast and pancreatic cancer cell lines. Cancer Biol Ther 16:714–723. 25996841 10.1080/15384047.2015.1026480 PMC4622877

[em22253-bib-0019] Dohda T , Khanh DG , Kamihira M , Iijima S . 2003 Essential role of cell‐cell interaction in primary hepatocyte cultures In: Yagasaki K, Miura Y, Hatori M, Nomura Y, editors. Animal Cell Technology: Basic & Applied Aspects: Proceedings of the Fifteenth Annual Meeting of the Japanese Association for Animal Cell Technology (JAACT), Fuchu, Japan, November 11–15, 2002. Dordrecht: Springer Netherlands pp. 347–351.

[em22253-bib-0020] Duncan AW , Taylor MH , Hickey RD , Hanlon Newell AE , Lenzi ML , Olson SB , Finegold MJ , Grompe M . 2010 The ploidy conveyor of mature hepatocytes as a source of genetic variation. Nature 467:707–710. 20861837 10.1038/nature09414 PMC2967727

[em22253-bib-0021] Duncan AW , Hanlon Newell AE , Bi W , Finegold MJ , Olson SB , Beaudet AL , Grompe M . 2012 Aneuploidy as a mechanism for stress‐induced liver adaptation. J Clin Investig 122:3307–3315. 22863619 10.1172/JCI64026 PMC3428097

[em22253-bib-0022] Elaut G , Henkens T , Papeleu P , Snykers S , Vinken M , Vanhaecke T , Rogiers V . 2006 Molecular mechanisms underlying the dedifferentiation process of isolated hepatocytes and their cultures. Curr Drug Metab 7:629–660. 16918317 10.2174/138920006778017759

[em22253-bib-0023] European Commission . 2009 Regulation (EC) No 1223/2009 of the European Parliament and of the Council of 30 November 2009 on cosmetic products. Offic J Eur Union 342:59–209.

[em22253-bib-0024] Gallagher EP , Wienkers LC , Stapleton PL , Kunze KL , Eaton DL . 1994 Role of human microsomal and human complementary DNA‐expressed cytochromes P4501A2 and P4503A4 in the bioactivation of aflatoxin B1. Cancer Res 54:101–108. 8261428

[em22253-bib-0025] Gerlyng P , Abyholm A , Grotmol T , Erikstein B , Huitfeldt HS , Stokke T , Seglen PO . 1993 Binucleation and polyploidization patterns in developmental and regenerative rat liver growth. Cell Prolif 26:557–565. 9116122 10.1111/j.1365-2184.1993.tb00033.x

[em22253-bib-0026] Glatt HR , Billings R , Platt KL , Oesch F . 1981 Improvement of the correlation of bacterial mutagenicity with carcinogenicity of benzo(a)pyrene and four of its major metabolites by activation with intact liver cells instead of cell homogenate. Cancer Res 41:270–277. 7448766

[em22253-bib-0027] Godoy P , Hengstler JG , Ilkavets I , Meyer C , Bachmann A , Müller A , Tuschl G , Mueller SO , Dooley S . 2009 Extracellular matrix modulates sensitivity of hepatocytes to fibroblastoid dedifferentiation and transforming growth factor β‐induced apoptosis. Hepatology 49:2031–2043. 19274752 10.1002/hep.22880

[em22253-bib-0028] Gossen JA , De Leeuw WJF , Tan CHT , Zwarthoff EC , Berends F , Lohman PHM , Knook DL , Vijg J . 1989 Efficient rescue of integrated shuttle vectors from transgenic mice: A model for studying mutations in vivo. Proc Natl Acad Sci USA 86:7971–7975. 2530578 10.1073/pnas.86.20.7971 PMC298194

[em22253-bib-0029] Guidotti J , Brégerie O , Robert A , Debey P , Brechot C , Desdouets C . 2003 Liver cell polyploidization: a pivotal role for binuclear hepatocytes. J Biol Chem 278:19095–19101. 12626502 10.1074/jbc.M300982200

[em22253-bib-0030] Heflich RH , Neft RE . 1994 Genetic toxicity of 2‐acetylaminofluorene, 2‐aminofluorene and some of their metabolites and model metabolites. Mutat Res 318:73–174. 7521935 10.1016/0165-1110(94)90025-6

[em22253-bib-0031] Herzog N , Hansen M , Miethbauer S , Schmidtke K , Anderer U , Lupp A , Sperling S , Seehofer D , Damm G , Scheibner K , et al. 2016 Primary‐like human hepatocytes genetically engineered to obtain proliferation competence display hepatic differentiation characteristics in monolayer and organotypical spheroid cultures. Cell Biol Int 40:341–353. 26715207 10.1002/cbin.10574

[em22253-bib-0032] Hook EB . 1977 Exclusion of chromosomal mosaicism: tables of 90%, 95% and 99% confidence limits and comments on use. Am J Hum Genet 29:94–97. 835578 PMC1685228

[em22253-bib-0033] Hoorn AJW , Custer LL , Myhr BC , Brusick D , Gossen J , Vijg J . 1993 Detection of chemical mutagens using Muta™Mouse: a transgenic mouse model. Mutagenesis 8:7–10. 8450770 10.1093/mutage/8.1.7

[em22253-bib-0034] Huang MT , Miwa GT , Cronheim N , Lu AY . 1979 Rat liver cytosolic azoreductase. Electron transport properties and the mechanism of dicumarol inhibition of the purified enzyme. J Biol Chem 254:11223–11227. 91608

[em22253-bib-0035] Johnson TE , Umbenhauer DR , Galloway SM . 1996 Human liver S‐9 metabolic activation: Proficiency in cytogenetic assays and comparison with phenobarbital/β‐naphthoflavone or Aroclor 1254 induced rat S‐9. Environ Mol Mutagen 28:51–59. 8698047 10.1002/(SICI)1098-2280(1996)28:1<51::AID-EM8>3.0.CO;2-H

[em22253-bib-0036] Jossé R , Rogue A , Lorge E , Guillouzo A . 2012 An adaptation of the human HepaRG cells to the in vitro micronucleus assay. Mutagenesis 27:295–304. 22058015 10.1093/mutage/ger076

[em22253-bib-0037] Kennedy SW , Jones SP . 1994 Simultaneous measurement of cytochrome P4501A catalytic activity and total protein concentration with a fluorescence plate reader. Anal Biochem 222:217–223. 7856852 10.1006/abio.1994.1476

[em22253-bib-0038] Kennedy SW , Jones SP , Bastien LJ . 1995 Efficient analysis of cytochrome P4501A catalytic activity, porphyrins, and total proteins in chicken embryo hepatocyte cultures with a fluorescence plate reader. Anal Biochem 226:362–370. 7793639 10.1006/abio.1995.1237

[em22253-bib-0039] Kim JH , Stansbury KH , Walker NJ , Trush MA , Strickland PT , Sutter TR . 1998 Metabolism of benzo[a]pyrene and benzo[a]pyrene‐7,8‐diol by human cytochrome P450 1B1. Carcinogenesis 19:1847–1853. 9806168 10.1093/carcin/19.10.1847

[em22253-bib-0040] Kirkland D , Kasper P , Müller L , Corvi R , Speit G . 2008 Recommended lists of genotoxic and non‐genotoxic chemicals for assessment of the performance of new or improved genotoxicity tests: a follow‐up to an ECVAM workshop. Mutat Res 653:99–108. 18539078 10.1016/j.mrgentox.2008.03.008

[em22253-bib-0041] Kirkland D , Pfuhler S , Tweats D , Aardema M , Corvi R , Darroudi F , Elhajouji A , Glatt H , Hastwell P , Hayashi M , et al. 2007 How to reduce false positive results when undertaking in vitro genotoxicity testing and thus avoid unnecessary follow‐up animal tests: report of an ECVAM Workshop. Mutat Res 628:31–55. 17293159 10.1016/j.mrgentox.2006.11.008

[em22253-bib-0042] Kirkland DJ , Marshall RR , McEnaney S , Bidgood J , Rutter A , Mullineux S . 1989 Aroclor‐1254‐induced rat‐liver S9 causes chromosomal aberrations in CHO cells but not human lymphocytes: a role for active oxygen? Mutat Res 214:115–122. 2505066 10.1016/0027-5107(89)90204-2

[em22253-bib-0043] Klaunig JE , Goldblatt PJ , Hinton DE . 1981 Mouse liver cell culture I. Hepatocyte isolation. In Vitro 17:913–925. 6273298 10.1007/BF02618288

[em22253-bib-0044] Kratochwil NA , Meille C , Fowler S , Klammers F , Ekiciler A , Molitor B , Simon S , Walter I , McGinnis C , Walther J , et al. 2017 Metabolic profiling of human long‐term liver models and hepatic clearance predictions from in vitro data using nonlinear mixed‐effects modeling. AAPS J 19:534–550. 28050713 10.1208/s12248-016-0019-7

[em22253-bib-0045] Kreamer BL , Staecker JL , Sawada N . 1986 Use of a low‐speed, iso‐density percoll centrifugation method to increase the viability of isolated rat hepatocyte preparations. In Vitro 22:201–211. 10.1007/BF02623304 2871008

[em22253-bib-0046] Kruglov EA , Jain D , Dranoff JA . 2002 Isolation of primary rat liver fibroblasts. J Investig Med 50:179–184. 10.2310/6650.2002.33431 12033282

[em22253-bib-0047] Ku WW , Bigger A , Brambilla G , Glatt H , Gocke E , Guzzie PJ , Hakura A , Honma M , Martus H , Obach RS , et al. 2007 Strategy for genotoxicity testing‐metabolic considerations. Mutat Res 627:59–77. 17141553 10.1016/j.mrgentox.2006.10.004

[em22253-bib-0048] Lambert CB , Spire C , Renaud M , Claude N , Guillouzo A . 2009 Reproducible chemical‐induced changes in gene expression profiles in human hepatoma HepaRG cells under various experimental conditions. Toxicol Vitro 23:466–475. 10.1016/j.tiv.2008.12.018 19159669

[em22253-bib-0049] Lambert IB , Singer TM , Boucher SE , Douglas GR . 2005 Detailed review of transgenic rodent mutation assays. Mutat Res 590:1–280. 16081315 10.1016/j.mrrev.2005.04.002

[em22253-bib-0050] Li P , Li J , Li M , Gong J , He K . 2014 An efficient method to isolate and culture mouse Kupffer cells. Immunol Lett 158:52–56. 24333337 10.1016/j.imlet.2013.12.002

[em22253-bib-0051] Liu F , Lin Y , Li Z , Ma X , Han Q , Liu Y , Zhou Q , Liu J , Li R , Li J , et al. 2014 Glutathione S‐transferase A1 (GSTA1) release, an early indicator of acute hepatic injury in mice. Food Chem Toxicol 71:225–230. 24964013 10.1016/j.fct.2014.06.011

[em22253-bib-0052] Livak KJ , Schmittgen TD . 2001 Analysis of relative gene expression data using real‐time quantitative PCR and the 2‐ΔΔCT method. Methods 25:402–408. 11846609 10.1006/meth.2001.1262

[em22253-bib-0053] Lnenikova K , Skalova L , Stuchlikova L , Szotakova B , Matouakova P . 2018 Induction of xenobiotic‐metabolizing enzymes in hepatocytes by beta‐naphthoflavone: time‐dependent changes in activities, protein and mRNA levels. Acta Pharm 68:75–85. 29453911 10.2478/acph-2018-0005

[em22253-bib-0054] Lorge E , Moore MM , Clements J , O'Donovan M , Fellows MD , Honma M , Kohara A , Galloway S , Armstrong MJ , Thybaud V , et al. 2016 Standardized cell sources and recommendations for good cell culture practices in genotoxicity testing. Mutat Res 809:1–15. 27692294 10.1016/j.mrgentox.2016.08.001

[em22253-bib-0055] Lübberstedt M , Müller‐Vieira U , Mayer M , Biemel KM , Knöspel F , Knobeloch D , Nüssler AK , Gerlach JC , Zeilinger K . 2011 HepaRG human hepatic cell line utility as a surrogate for primary human hepatocytes in drug metabolism assessment in vitro. J Pharmacol Toxicol Methods 63:59–68. 20460162 10.1016/j.vascn.2010.04.013

[em22253-bib-0056] Luijten M , Zwart EP , Dollé MET , de Pooter M , Cox JA , White PA , van Benthem J . 2016 Evaluation of the LacZ reporter assay in cryopreserved primary hepatocytes for in vitro genotoxicity testing. Environ Mol Mutagen 57:643–655. 27859631 10.1002/em.22063

[em22253-bib-0057] Madle E , Tiedemann G , Madle S , Ott A , Kaufmann G . 1986 Comparison of S9 mix and hepatocytes as external metabolizing systems in mammalian cell cultures: cytogenetic effects of 7,12‐dimethylbenzanthracene and aflatoxin B1. Environ Mutagen 8:423–437. 3086074 10.1002/em.2860080311

[em22253-bib-0058] Mathijs K , Kienhuis AS , Brauers KJJ , Jennen DGJ , Lahoz A , Kleinjans JCS , van Delft JHM . 2009 Assessing the metabolic competence of sandwich‐cultured mouse primary hepatocytes. Drug Metab Dispos 37:1305–1311. 19251822 10.1124/dmd.108.025775

[em22253-bib-0059] McMahon TF , Cunningham ML , Prival MJ . 1991 Mutagenicity of methylazoxymethanol acetate in the presence of alcohol dehydrogenase, aldehyde dehydrogenase, and rat liver microsomes in *Salmonella typhimurium* his G46. Environ Mol Mutagen 18:151–156. 1915309 10.1002/em.2850180302

[em22253-bib-0060] Miller GE , Brabec MJ , Kulkarni AP . 1986 Mutagen activation of 1,2‐dibromo‐3‐chloropropane by cytosolic glutathione s‐transferases and microsomal enzymes. J Toxicol Environ Health 19:503–518. 3537323 10.1080/15287398609530948

[em22253-bib-0061] Møller P , Wallin H . 2000 Genotoxic hazards of azo pigments and other colorants related to 1‐phenylazo‐2‐hydroxynaphthalene. Mutat Res 462:13–30. 10648921 10.1016/s1383-5742(99)00090-3

[em22253-bib-0062] Nelson DR , Zeldin DC , Hoffman SMG , Maltais LJ , Wain HM , Nebert DW . 2004 Comparison of cytochrome P450 (CYP) genes from the mouse and human genomes, including nomenclature recommendations for genes, pseudogenes and alternative‐splice variants. Pharmacogenetics 14:1–18. 15128046 10.1097/00008571-200401000-00001

[em22253-bib-0063] Nüsse M , Beisker W , Kramer J , Miller BM , Schreiber GA , Viaggi S , Weller EM , Wessels JM . 1994 Chapter 9 measurement of micronuclei by flow cytometry. Methods Cell Biol 42:149–158. 7533237 10.1016/s0091-679x(08)61072-9

[em22253-bib-0064] OECD . 2016a. OECD guidelines for testing of chemicals, section 4, test no. 473: in vitro mammalian chromosome aberration test. OECD Publishing, Paris.

[em22253-bib-0065] OECD . 2016b OECD guidelines for the testing of chemicals, section 4, test no. 476: in vitro mammalian cell gene mutation tests using the Hprt and Xprt genes. OECD Publishing, Paris.

[em22253-bib-0066] OECD . 2016c OECD guidelines for the testing of chemicals, section 4, test no. 487: in vitro mammalian cell micronucleus test. OECD Publishing, Paris.

[em22253-bib-0067] OECD . 2016d OECD guidelines for the testing of chemicals section 4, test no. 490: in vitro mammalian cell gene mutation tests using the thymidine kinase gene. OECD Publishing, Paris.

[em22253-bib-0068] OECD . 2013 OECD guidelines for the testing of chemicals, section 4, test no. 488: Transgenic rodent somatic and germ cell gene mutation assays. OECD Publishing, Paris.

[em22253-bib-0069] OECD . 2005 Guidance document on the validation and international acceptance of new or updated test methods for hazard assessment, No. 14, OECD Publishing, Paris.

[em22253-bib-0070] Radominska‐Pandya A , Czernik PJ , Little JM , Battaglia E , Mackenzie PI . 1999 Structural and functional studies of UDP‐glucuronosyltransferases. Drug Metab Rev 31:817–899. 10575553 10.1081/dmr-100101944

[em22253-bib-0071] Rowe C , Goldring CEP , Kitteringham NR , Jenkins RE , Lane BS , Sanderson C , Elliott V , Platt V , Metcalfe P , Park BK . 2010 Network analysis of primary hepatocyte dedifferentiation using a shotgun proteomics approach. J Proteome Res 9:2658–2668. 20373825 10.1021/pr1001687

[em22253-bib-0072] Roymans D , Annaert P , Van Houdt J , Weygers A , Noukens J , Sensenhauser C , Silva J , Van Looveren C , Hendrickx J , Mannens G , et al. 2005 Expression and induction potential of cytochromes p450 in human cryopreserved hepatocytes. Drug Metab Dispos 33:1004–1016. 15802389 10.1124/dmd.104.003046

[em22253-bib-0073] Schut HAJ , Snyderwine EG . 1999 DNA adducts of heterocyclic amine food mutagens: Implications for mutagenesis and carcinogenesis. Carcinogenesis 20:353–368. 10190547 10.1093/carcin/20.3.353

[em22253-bib-0074] Seglen PO . 1976 Preparation of isolated rat liver cells. Methods Cell Biol 13:29–83. 177845 10.1016/s0091-679x(08)61797-5

[em22253-bib-0075] Sohn OS , Fiala ES , Requeijo SP , Weisburger JH , Gonzalez FJ . 2001 Differential effects of CYP2E1 status on the metabolic activation of the colon carcinogens azoxymethane and methylazoxymethanol. Cancer Res 61:8435–8440. 11731424

[em22253-bib-0076] Storer RD , Kraynak AR , McKelvey TW , Elia MC , Goodrow TL , DeLuca JG . 1997 The mouse lymphoma L5178Y Tk(+/−) cell line is heterozygous for a codon 170 mutation in the p53 tumor suppressor gene. Mutat Res Fundam Mol Mech Mutagen 373:157–165. 10.1016/s0027-5107(96)00227-8 9042396

[em22253-bib-0077] Tice RR , Austin CP , Kavlock RJ , Bucher JR . 2013 Improving the human hazard characterization of chemicals: a Tox21 update. Environ Health Perspect 121:756–765. 23603828 10.1289/ehp.1205784 PMC3701992

[em22253-bib-0078] Truisi GL , Consiglio ED , Parmentier C , Savary CC , Pomponio G , Bois F , Lauer B , Jossé R , Hewitt PG , Mueller SO , et al. 2015 Understanding the biokinetics of ibuprofen after single and repeated treatments in rat and human in vitro liver cell systems. Toxicol Lett 233:172–186. 25578229 10.1016/j.toxlet.2015.01.006

[em22253-bib-0079] Tuschl G , Mueller SO . 2006 Effects of cell culture conditions on primary rat hepatocytes‐cell morphology and differential gene expression. Toxicology 218:205–215. 16337326 10.1016/j.tox.2005.10.017

[em22253-bib-0080] van Bladeren PJ , Breimer DD , Rotteveel‐Smijs GMT , Mohn GR . 1980 Mutagenic activation of dibromomethane and diiodomethane by mammalian microsomes and glutathione S‐transferases. Mutat Res 74:341–346. 7010125 10.1016/0165-1161(80)90192-2

[em22253-bib-0081] Van Eyken P , Sciot R , Damme B , Wolf‐Peeters C , Desmet V . 1987 Keratin immunohistochemistry in normal human liver. Cytokeratin pattern of hepatocytes, bile ducts and acinar gradient. Vichows Archiv A Pathol Anat 412:63–72. 10.1007/BF00750732 2446418

[em22253-bib-0082] Wang Z , Luo X , Anene‐Nzelu C , Yu Y , Hong X , Singh NH , Xia L , Liu S , Yu H . 2015 HepaRG culture in tethered spheroids as an in vitro three‐dimensional model for drug safety screening. J Appl Toxicol 35:909–917. 25512232 10.1002/jat.3090

[em22253-bib-0083] Wells MJ , Hatton MWC , Hewlett B , Podor TJ , Sheffield WP , Blajchman MA . 1997 Cytokeratin 18 is expressed on the hepatocyte plasma membrane surface and interacts with thrombin‐antithrombin complexes. J Biol Chem 272:28574–28581. 9353322 10.1074/jbc.272.45.28574

[em22253-bib-0084] White PA , Douglas GR , Gingerich J , Parfett C , Shwed P , Seligy V , Soper L , Berndt L , Bayley J , Wagner S , et al. 2003 Development and characterization of a stable epithelial cell line from MutaMouse lung. Environ Mol Mutagen 42:166–184. 14556224 10.1002/em.10185

[em22253-bib-0085] Xie H , Yasar U , Lundgren S , Griskevicius L , Terelius Y , Hassan M , Rane A . 2003 Role of polymorphic human CYP2B6 in cyclophosphamide bioactivation. Pharmacogenomics J 3:53–61. 12629583 10.1038/sj.tpj.6500157

[em22253-bib-0086] Yamazaki H , Inui Y , Yun C , Guengerich FP , Shimada T . 1992 Cytochrome P450 2E1 and 2A6 enzymes as major catalysts for metabolic activation of N‐nitrosodialkylamines and tobacco‐related nitrosamines in human liver microsomes. Carcinogenesis 13:1789–1794. 1423839 10.1093/carcin/13.10.1789

[em22253-bib-0087] Yang J , Liao M , Shou M , Jamei M , Yeo KR , Tucker GT , Rostami‐Hodjegan A . 2008 Cytochrome P450 turnover: Regulation of synthesis and degradation, methods for determining rates, and implications for the prediction of drug interactions. Curr Drug Metab 9:384–393. 18537575 10.2174/138920008784746382

[em22253-bib-0088] Yokoi Y , Namihisa T , Kuroda H , Komatsu I , Miyazaki A , Watanabe S , Usui K . 1984 Immunocytochemical detection of desmin in fat‐storing cells (Ito cells). Hepatology 4:709–714. 6204917 10.1002/hep.1840040425

[em22253-bib-0089] Zeiger E . 2010 Historical perspective on the development of the genetic toxicity test battery in the United States. Environ Mol Mutagen 51:781–791. 20740645 10.1002/em.20602

[em22253-bib-0090] Zwart EP , Schaap MM , van den Dungen MW , Braakhuis HM , White PA , Steeg HV , Benthem JV , Luijten M . 2012 Proliferating primary hepatocytes from the pUR288 lacZ plasmid mouse are valuable tools for genotoxicity assessment in vitro. Environ Mol Mutagen 53:376–383. 10.1002/em.21700 22619112

