# Supplementary material for: The development and prevalidation of an in vitro mutagenicity assay based on MutaMouse primary hepatocytes, Part I: Isolation, structural, genetic, and biochemical characterization
Source: Environ Mol Mutagen. 2018 Dec 27;60(4):331–47. doi: 10.1002/em.22253 (PMC6590113; doi:10.1002/em.22253)
Supplement: Supplementary file 1 — Supplementary Table I: Primary and secondary antibodies used for immunocytochemical analyses Supplementary Table II: Settings for the electrospray ion sources used for the acquisition of testosterone, 16β‐hydroxytestosterone, 6β‐hydroxytestosterone, and 7‐hydroxycoumarin data with a Waters Xevo TQMS, and acquisition of 7‐hydroxycoumarin sulphate and 7‐hydroxycoumarin glucuronide data with a Waters TQS. Supplementary Table III: Mass spectrometric detection parameters optimized via Multiple Reaction Monitoring (MRM) methods using Waters QuanOptimise. ES‐ electrospray ionization. + indicates positive ion. Supplementary Table IV: UPLC gradient profile used for analysis of testosterone, 16β‐hydroxytestosterone, 6β‐hydroxytestosterone and 7‐hydroxycoumarin. Supplementary Table V: UPLC gradient profile used for analysis of 7‐hydroxycoumarin sulphate and 7‐hydroxycoumarin glucuronide Supplementary Table VI: Genes included in the Qiagen Mouse Drug Metabolism RT2 profiler PCR array. Genes shaded in green are housekeeping genes for normalization and genes shaded in grey are qPCR controls. Supplementary Table VII: The fold‐changes in gene expression in MutaMouse primary hepatocytes over time for 84 murine metabolism. Expression measured using the Qiagen Mouse Drug Metabolism RT2 profiler PCR array. Red‐shaded cells indicate a significant fold‐increase, whereas green‐shaded cells indicate a significant fold‐decrease (P ≤ 0.05). Grey shading indicates lack of signal or a Ct value above the cut‐off of 35. Supplementary Figure 1: Histogram and dot plots of primary MutaMouse hepatocyte nuclei illustrating the gates used to discriminate bead and nuclei populations from noise and spurious events. Events displayed and scored (panel A) were required to fall within the FL1 range (panel B), the light scatter region (panel C), the SSC vs FSC region (panel D), and the region that excludes doublets (panel E). The resulting FSC versus FL1 dot plot (panel A) displays a distinct bead population [file EM-60-331-s001.docx]

**Supplementary Tables and Figures:**

**Supplementary Table I.** Primary and secondary antibodies used for immunocytochemical analyses

| Antibody | Target | Host | Clonality | Conjugate | Dilution | Positive control cell line |
| --- | --- | --- | --- | --- | --- | --- |
| *Primary antibodies* | | | | | | |
| Abcam ab19194 | Mouse serum albumin | Goat | Polyclonal | No | 1/200 | HepG2 |
| Abcam ab14047 | Human cytokeratin 18 (mouse reactivity) | Chicken | Polyclonal | No | 1/100 | HepG2 |
| Abcam ab53119 | Human cytokeratin 19 (mouse reactivity) | Rabbit | Polyclonal | No | 1/100 | HepG2 |
| Cell Signaling Technology 9854 | Human vimentin (mouse reactivity) | Rabbit | Monoclonal | Alexa Fluor® 488 | 1/500 | A549 |
| Abcam ab15200 | Human desmin (mouse reactivity) | Rabbit | Polyclonal | No | 1/100 | C2C12 |
| Abcam ab60343 | Mouse F4/80 | Rat | Monoclonal | FITC | 1/100 | RAW 264.7 |
| *Secondary antibodies* | | | | | | |
| ThermoFisher Scientific A11058 | Goat IgG | Donkey | Polyclonal | Alexa Fluor® 594 | 1/2000 | NA |
| ThermoFisher Scientific A11039 | Chicken IgY | Goat | Polyclonal | Alexa Fluor® 488 | 1/2000 | NA |
| ThermoFisher Scientific A21441 | Rabbit IgG | Chicken | Polyclonal | Alexa Fluor® 488 | 1/2000 | NA |

**Supplementary Table II**: Settings for the electrospray ion sources used for the acquisition of testosterone, 16β-hydroxytestosterone, 6β-hydroxytestosterone, and 7-hydroxycoumarin data with a Waters Xevo TQMS, and acquisition of 7-hydroxycoumarin sulphate and 7-hydroxycoumarin glucuronide data with a Waters TQS.

| Parameter | Setting Xevo TQMS | Setting TQS |
| --- | --- | --- |
| Capillary voltage (kV) | 3.5 | 0.7 |
| Source temperature (°C) | 150 | 150 |
| Desolvation gas temperature (°C) | 500 | 650 |
| Desolvation gas flow (L/h) | 1000 | 1200 |
| Cone gas flow (L/h) | 100 | 150 |
| Collision gas flow (mL/min) | 0.17 | 0.15 |

**Supplementary Table III**: Mass spectrometric detection parameters optimized via Multiple Reaction Monitoring (MRM) methods using Waters QuanOptimise. ES- electrospray ionization. + indicates positive ion.

| Compound ID | Ionisation mode | Transition | Cone voltage (V) | Collision energy (eV) |
| --- | --- | --- | --- | --- |
| Testosterone | ES+ | 289.53 > 109.08 | 42 | 28 |
| 16β-hydroxytestosterone | ES+ | 305.17 > 96.91 | 28 | 22 |
| 6β-hydroxytestosterone | ES+ | 305.14 > 268.98 | 28 | 16 |
| 7-hydroxycoumarin | ES+ | 162.98 > 107.02 | 28 | 22 |
| 7-hydroxycoumarin sulphate | ES- | 240.81 > 160.85 | 35 | 15 |
| 7-hydroxycoumarin glucuronide | ES+ | 338.80 > 162.90 | 35 | 15 |
| Diclofenac | ES+ | 296.05 > 214.14 | 22 (TQMS)  10 (TQS) | 32 (TQMS)  30 (TQS) |
| Reserpine | ES+ | 609.37 > 195.09 | 46 (TQMS)  66 (TQS) | 36 (TQMS)  34 (TQS) |

**Supplementary Table IV**: UPLC gradient profile used for analysis of testosterone, 16β-hydroxytestosterone, 6β-hydroxytestosterone and 7-hydroxycoumarin.

| Parameter | Setting | |
| --- | --- | --- |
| Column | Kinetex XB-C18 100A 50 x 2.1 mm, 2.6 µm | |
| Column temperature | 40 °C | |
| Flow rate | 0.7 mL/min | |
| Injection volume | 1 µL (testosterone, 16β-hydroxytestosterone)  2 µL (6β-hydroxytestosterone, 7-hydroxycoumarin) | |
| Mobile phase A | 0.01% Formic acid in water (v/v) | |
| Mobile phase B | 0.01% Formic acid in acetonitrile (v/v) | |
| Gradient profile | Time (minutes) | Mobile phase B (%) |
|  | 0.00 | 5 |
|  | 0.20 | 5 |
|  | 1.20 | 95 |
|  | 1.80 | 95 |
|  | 1.85 | 5 |
|  | 2.00 | 5 |
| Retention time | Testosterone | 1.05 minutes |
|  | 16β-hydroxytestosterone | 0.91 minutes |
|  | 6β-hydroxytestosterone | 0.85 minutes |
|  | 7-hydroxycoumarin | 0.76 minutes |
|  | Diclofenac | 1.16 minutes |
|  | Reserpine | 0.89 minutes |

**Supplementary Table V**: UPLC gradient profile used for analysis of 7-hydroxycoumarin sulphate and 7-hydroxycoumarin glucuronide

| Parameter | Setting | |
| --- | --- | --- |
| Column | Acquite BEH-C18 100A 50 x 2.1 mm, 1.7 µm | |
| Column temperature | 40 °C | |
| Flow rate | 0.8 mL/min | |
| Injection volume | 2 µL | |
| Mobile phase A | 0.1% Formic acid in water (v/v) | |
| Mobile phase B | 0.1% Formic acid in acetonitrile (v/v) | |
| Gradient profile | Time (minutes) | Mobile phase B (%) |
|  | 0.00 | 2 |
|  | 0.25 | 2 |
|  | 1.00 | 98 |
|  | 1.50 | 98 |
|  | 1.60 | 2 |
| Retention time | 7-hydroxycoumarin glucuronide | 0.66 minutes |
|  | 7-hydroxycoumarin sulphate | 0.73 minutes |
|  | Diclofenac | 1.04 minutes |
|  | Reserpine | 0.84 minutes |

**Supplementary Table VI**: Genes included in the Qiagen Mouse Drug Metabolism RT2 profiler PCR array. Genes shaded in green are housekeeping genes for normalization and genes shaded in grey are qPCR controls.

| Symbol | Description |
| --- | --- |
| Abcb1a | ATP-binding cassette, sub-family B (MDR/TAP), member 1A |
| Abcb1b | ATP-binding cassette, sub-family B (MDR/TAP), member 1B |
| Abcb4 | ATP-binding cassette, sub-family B (MDR/TAP), member 4 |
| Abcc1 | ATP-binding cassette, sub-family C (CFTR/MRP), member 1 |
| Aoc1 | Amiloride binding protein 1 (amine oxidase, copper-containing) |
| Adh1 | Alcohol dehydrogenase 1 (class I) |
| Adh4 | Alcohol dehydrogenase 4 (class II), pi polypeptide |
| Adh5 | Alcohol dehydrogenase 5 (class III), chi polypeptide |
| Ahr | Aryl-hydrocarbon receptor |
| Alad | Aminolevulinate, delta-, dehydratase |
| Aldh1a1 | Aldehyde dehydrogenase family 1, subfamily A1 |
| Alox12 | Arachidonate 12-lipoxygenase |
| Alox15 | Arachidonate 15-lipoxygenase |
| Alox5 | Arachidonate 5-lipoxygenase |
| Apoe | Apolipoprotein E |
| Arnt | Aryl hydrocarbon receptor nuclear translocator |
| Asna1 | ArsA arsenite transporter, ATP-binding, homolog 1 (bacterial) |
| Blvra | Biliverdin reductase A |
| Blvrb | Biliverdin reductase B (flavin reductase (NADPH)) |
| Ces1g | Carboxylesterase 1G |
| Ces2c | Carboxylesterase 2C |
| Chst1 | Carbohydrate (keratan sulfate Gal-6) sulfotransferase 1 |
| Comt | Catechol-O-methyltransferase |
| Cyb5r3 | Cytochrome b5 reductase 3 |
| Cyp11b2 | Cytochrome P450, family 11, subfamily b, polypeptide 2 |
| Cyp17a1 | Cytochrome P450, family 17, subfamily a, polypeptide 1 |
| Cyp19a1 | Cytochrome P450, family 19, subfamily a, polypeptide 1 |
| Cyp1a1 | Cytochrome P450, family 1, subfamily a, polypeptide 1 |
| Cyp1a2 | Cytochrome P450, family 1, subfamily a, polypeptide 2 |
| Cyp27b1 | Cytochrome P450, family 27, subfamily b, polypeptide 1 |
| Cyp2c29 | Cytochrome P450, family 2, subfamily c, polypeptide 29 |
| Cyp2e1 | Cytochrome P450, family 2, subfamily e, polypeptide 1 |
| Cyp3a11 | Cytochrome P450, family 3, subfamily a, polypeptide 11 |
| Cyp3a44 | Cytochrome P450, family 3, subfamily a, polypeptide 44 |
| Cyp4b1 | Cytochrome P450, family 4, subfamily b, polypeptide 1 |
| Ephx1 | Epoxide hydrolase 1, microsomal |
| Ephx2 | Epoxide hydrolase 2, cytoplasmic |
| Faah | Fatty acid amide hydrolase |
| Fbp1 | Fructose bisphosphatase 1 |
| Gad1 | Glutamic acid decarboxylase 1 |
| Gad2 | Glutamic acid decarboxylase 2 |
| Gckr | Glucokinase regulatory protein |
| Ggt1 | Gamma-glutamyltransferase 1 |
| Gpi1 | Glucose phosphate isomerase 1 |
| Gpx1 | Glutathione peroxidase 1 |
| Gpx2 | Glutathione peroxidase 2 |
| Gpx3 | Glutathione peroxidase 3 |
| Gpx5 | Glutathione peroxidase 5 |
| Gsr | Glutathione reductase |
| Gsta1 | Glutathione S-transferase, alpha 1 (Ya) |
| Gsta3 | Glutathione S-transferase, alpha 3 |
| Gsta4 | Glutathione S-transferase, alpha 4 |
| Gstm1 | Glutathione S-transferase, mu 1 |
| Gstm2 | Glutathione S-transferase, mu 2 |
| Gstm3 | Glutathione S-transferase, mu 3 |
| Gstm4 | Glutathione S-transferase, mu 4 |
| Gstm5 | Glutathione S-transferase, mu 5 |
| Gstp1 | Glutathione S-transferase, pi 1 |
| Gstt1 | Glutathione S-transferase, theta 1 |
| Gstz1 | Glutathione transferase zeta 1 (maleylacetoacetate isomerase) |
| Hk2 | Hexokinase 2 |
| Hsd17b1 | Hydroxysteroid (17-beta) dehydrogenase 1 |
| Hsd17b2 | Hydroxysteroid (17-beta) dehydrogenase 2 |
| Hsd17b3 | Hydroxysteroid (17-beta) dehydrogenase 3 |
| Lpo | Lactoperoxidase |
| Mgst1 | Microsomal glutathione S-transferase 1 |
| Mgst2 | Microsomal glutathione S-transferase 2 |
| Mgst3 | Microsomal glutathione S-transferase 3 |
| Mpo | Myeloperoxidase |
| Mt2 | Metallothionein 2 |
| Mt3 | Metallothionein 3 |
| Mthfr | 5,10-methylenetetrahydrofolate reductase |
| Nat1 | N-acetyl transferase 1 |
| Nat2 | N-acetyltransferase 2 (arylamine N-acetyltransferase) |
| Nos3 | Nitric oxide synthase 3, endothelial cell |
| Nqo1 | NAD(P)H dehydrogenase, quinone 1 |
| Pklr | Pyruvate kinase liver and red blood cell |
| Pkm | Pyruvate kinase, muscle |
| Pon1 | Paraoxonase 1 |
| Pon2 | Paraoxonase 2 |
| Pon3 | Paraoxonase 3 |
| Snn | Stannin |
| Srd5a1 | Steroid 5 alpha-reductase 1 |
| Srd5a2 | Steroid 5 alpha-reductase 2 |
| Actb | Actin, beta |
| B2m | Beta-2 microglobulin |
| Gapdh | Glyceraldehyde-3-phosphate dehydrogenase |
| Gusb | Glucuronidase, beta |
| Hsp90ab1 | Heat shock protein 90 alpha (cytosolic), class B member 1 |
| MGDC | Mouse Genomic DNA Contamination |
| RTC | Reverse Transcription Control |
| PPC | Positive PCR Control |

**Supplementary Table VII.** The fold-changes in gene expression in MutaMouse primary hepatocytes over time for 84 murine metabolism. Expression measured using the Qiagen Mouse Drug Metabolism RT2 profiler PCR array. Red-shaded cells indicate a significant fold-increase, whereas green-shaded cells indicate a significant fold-decrease (p≤0.05). Grey shading indicates lack of signal or a C_t_ value above the cut-off of 35.

|  | 0 h | 2 h | 8 h | 24 h | 48 h |
| --- | --- | --- | --- | --- | --- |
| Abcb1a | 1.00 | **1.73** | **3.31** | **7.70** | **2.07** |
| Abcb1b | 1.00 | **20.46** | **190.96** | **5764.58** | **1474.46** |
| Abcb4 | 1.00 | 1.05 | 0.69 | **0.23** | **0.08** |
| Abcc1 | 1.00 | **2.93** | **7.37** | **15.25** | **13.59** |
| Aoc1 | 1.00 | 0.70 | ND^a^ | ND | 0.27 |
| Adh1 | 1.00 | 1.16 | **0.77** | **0.04** | **0.02** |
| Adh4 | 1.00 | 0.97 | **0.22** | **0.16** | **0.13** |
| Adh5 | 1.00 | 1.29 | 0.94 | **0.91** | **0.48** |
| Ahr | 1.00 | **1.93** | **1.78** | **2.56** | 0.86 |
| Alad | 1.00 | 1.02 | **0.62** | 0.78 | **0.33** |
| Aldh1a1 | 1.00 | 0.89 | 0.59 | **2.74** | 1.69 |
| Alox12 | 1.00 | 2.64 | 0.45 | 1.12 | **2.49** |
| Alox15 | 1.00 | 2.43 | 2.62 | 3.77 | 0.97 |
| Alox5 | 1.00 | 1.19 | **3.27** | **6.09** | **10.46** |
| Apoe | 1.00 | 1.04 | **0.79** | **0.49** | **0.13** |
| Arnt | 1.00 | **1.70** | 1.43 | **2.95** | **2.12** |
| Asna1 | 1.00 | 1.19 | 1.36 | **5.23** | **3.74** |
| Blvra | 1.00 | 1.01 | **1.26** | **4.11** | **1.91** |
| Blvrb | 1.00 | 1.26 | **4.06** | **7.72** | 1.37 |
| Ces1g | 1.00 | 0.94 | 0.57 | **0.22** | **0.01** |
| Ces2c | 1.00 | 1.12 | 0.92 | **0.73** | **0.23** |
| Chst1 | 1.00 | 3.70 | 2.13 | **6.25** | 3.35 |
| Comt | 1.00 | 1.28 | 0.73 | 1.26 | 1.01 |
| Cyb5r3 | 1.00 | 1.16 | 1.17 | 1.27 | 0.45 |
| Cyp11b2 | ND | ND | ND | ND | ND |
| Cyp17a1 | 1.00 | 1.01 | **0.54** | **0.11** | **0.00** |
| Cyp19a1 | ND | ND | ND | ND | ND |
| Cyp1a1 | 1.00 | 6.43 | **16.66** | **4.06** | 1.77 |
| Cyp1a2 | 1.00 | 1.07 | **0.61** | **0.26** | **0.02** |
| Cyp27b1 | ND | ND | ND | ND | ND |
| Cyp2c29 | 1.00 | 1.11 | 0.96 | **0.33** | **0.01** |
| Cyp2e1 | 1.00 | 1.00 | **0.67** | **0.19** | **0.00** |
| Cyp3a11 | 1.00 | 1.05 | 0.75 | 0.24 | **0.00** |
| Cyp3a44 | 1.00 | 1.32 | 0.97 | **0.37** | **0.00** |
| Cyp4b1 | 1.00 | 1.07 | 1.19 | **1.74** | 0.70 |
| Ephx1 | 1.00 | 1.25 | 0.96 | **3.00** | **3.78** |
| Ephx2 | 1.00 | 0.83 | **0.61** | **0.26** | **0.20** |
| Faah | 1.00 | 0.89 | **0.55** | **0.34** | **0.16** |
| Fbp1 | 1.00 | 1.07 | **0.81** | **0.22** | 0.03 |
| Gad1 | 1.00 | 1.82 | 0.75 | 0.94 | 4.58 |
| Gad2 | 1.00 | ND | ND | ND | ND |
| Gckr | 1.00 | 1.04 | 0.90 | **0.51** | **0.02** |
| Ggt1 | 1.00 | 1.08 | ND | 1.48 | 1.35 |
| Gpi1 | 1.00 | **1.60** | **2.52** | **5.06** | **2.61** |
| Gpx1 | 1.00 | 1.57 | 1.05 | 1.03 | **0.49** |
| Gpx2 | 1.00 | 7.41 | 8.65 | **35.11** | **16.12** |
| Gpx3 | 1.00 | 1.91 | **9.84** | **126.35** | **65.21** |
| Gpx5 | 1.00 | ND | ND | ND | 1.35 |
| Gsr | 1.00 | 1.75 | **3.93** | **15.28** | **5.04** |
| Gsta1 | 1.00 | 43.34 | **2612.45** | **5323.62** | **471.67** |
| Gsta3 | 1.00 | 1.03 | **0.67** | **0.13** | **0.04** |
| Gsta4 | 1.00 | 1.28 | **1.48** | **1.58** | 0.83 |
| Gstm1 | 1.00 | 1.26 | **1.31** | 0.77 | **0.17** |
| Gstm2 | 1.00 | 1.24 | 1.24 | 0.88 | **0.20** |
| Gstm3 | 1.00 | 1.16 | 1.47 | **0.51** | **0.02** |
| Gstm4 | 1.00 | 1.17 | 0.76 | **0.30** | **0.14** |
| Gstm5 | 1.00 | 1.53 | **2.17** | **3.70** | **1.91** |
| Gstp1 | 1.00 | 1.33 | **8.73** | **15.65** | **4.82** |
| Gstt1 | 1.00 | 1.25 | 0.76 | **0.40** | **0.19** |
| Gstz1 | 1.00 | 0.99 | 0.72 | **0.18** | **0.05** |
| Hk2 | 1.00 | **5.16** | 1.83 | **15.81** | **29.01** |
| Hsd17b1 | 1.00 | 0.37 | **1.77** | **1.93** | 1.64 |
| Hsd17b2 | 1.00 | 1.12 | **0.77** | 0.90 | **0.51** |
| Hsd17b3 | 1.00 | **2.25** | 1.88 | ND | **0.20** |
| Lpo | 1.00 | ND | ND | ND | 2.52 |
| Mgst1 | 1.00 | 1.11 | 0.98 | 0.55 | **0.21** |
| Mgst2 | ND | ND | ND | ND | ND |
| Mgst3 | 1.00 | 1.26 | **1.47** | 2.20 | 1.08 |
| Mpo | 1.00 | 0.39 | 0.76 | 0.36 | 0.67 |
| Mt2 | 1.00 | **39.54** | **198.73** | **7.08** | 2.19 |
| Mt3 | 1.00 | **7.71** | **325.24** | **476.17** | **39.89** |
| Mthfr | 1.00 | 0.78 | 0.69 | **6.25** | 1.92 |
| Nat1 | 1.00 | 0.65 | **0.08** | **0.14** | **0.21** |
| Nat2 | 1.00 | 0.72 | 1.53 | **3.62** | 0.99 |
| Nos3 | 1.00 | 2.31 | 1.75 | 0.62 | 0.60 |
| Nqo1 | 1.00 | 1.14 | **7.19** | **5.74** | 0.95 |
| Pklr | 1.00 | 1.11 | 0.79 | 0.64 | 0.43 |
| Pkm | 1.00 | 1.58 | **3.98** | **21.31** | **24.49** |
| Pon1 | 1.00 | 1.06 | 0.67 | **0.33** | **0.06** |
| Pon2 | 1.00 | 1.12 | 0.99 | **3.95** | **2.30** |
| Pon3 | 1.00 | 1.19 | 0.90 | **2.40** | **1.93** |
| Snn | 1.00 | 0.91 | 1.97 | **8.02** | **20.58** |
| Srd5a1 | 1.00 | 1.01 | 0.39 | 0.66 | **0.20** |
| Srd5a2 | 1.00 | 1.19 | **0.41** | 1.47 | **1.73** |

^a^ ND, not determined


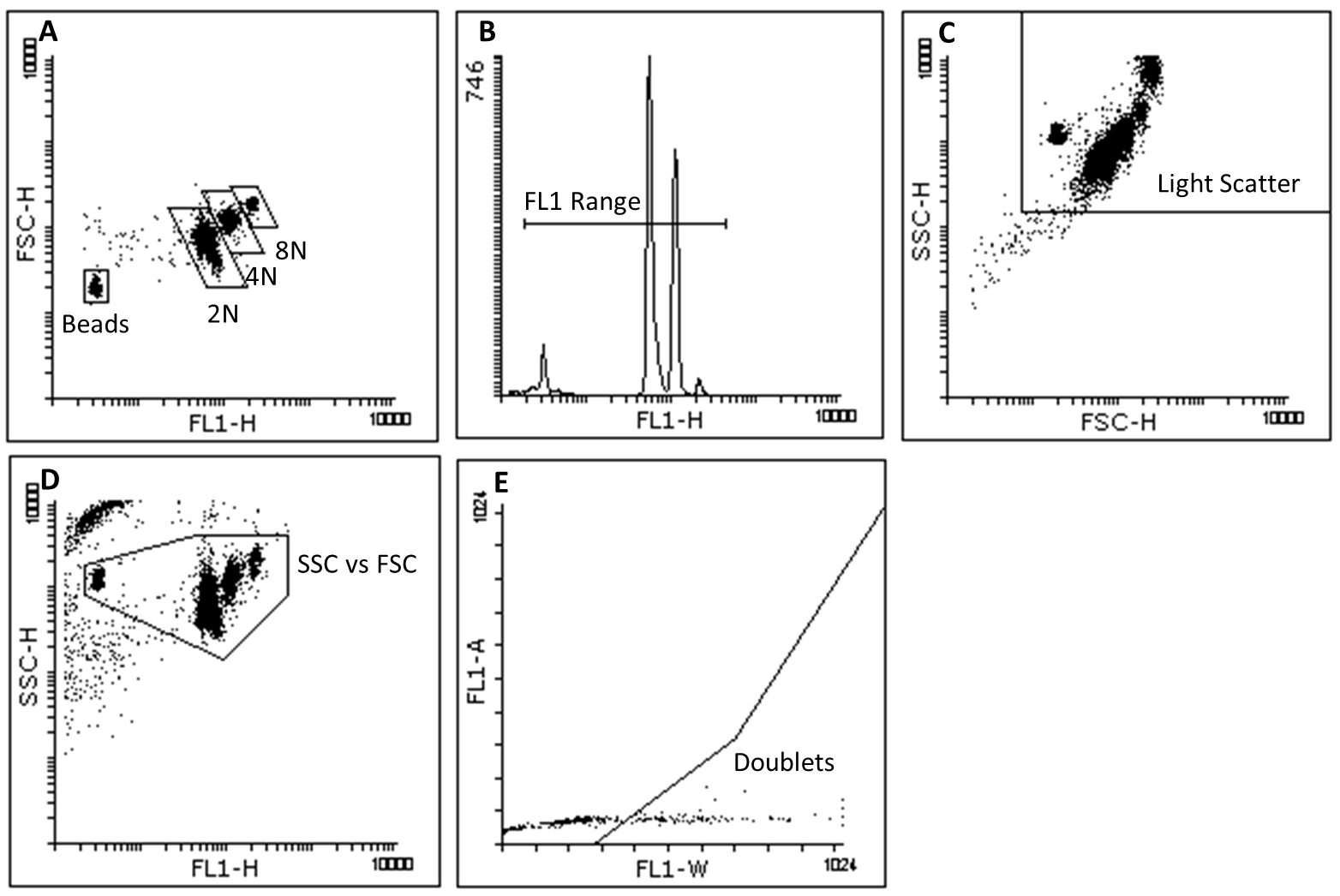


**Supplementary Figure 1**: Histogram and dot plots of primary MutaMouse hepatocyte nuclei illustrating the gates used to discriminate bead and nuclei populations from noise and spurious events. Events displayed and scored (panel A) were required to fall within the FL1 range (panel B), the light scatter region (panel C), the SSC vs FSC region (panel D), and the region that excludes doublets (panel E). The resulting FSC versus FL1 dot plot (panel A) displays a distinct bead population, as well as three populations representing 2n, 4n, and 8n nuclei.

**Supplementary Video 1.** Time-lapse imaging of MutaMouse primary hepatocytes in culture. The imaging begins 2 hours post-isolation and continues for 120 hours. Individual images were captured at 10 minute intervals at 10X magnification using bright-field imaging.
