# Supplementary material for: The development and prevalidation of an in vitro mutagenicity assay based on MutaMouse primary hepatocytes, Part I: Isolation, structural, genetic, and biochemical characterization
Source: Environ Mol Mutagen. 2018 Dec 27;60(4):331–47. doi: 10.1002/em.22253 (PMC6590113; doi:10.1002/em.22253)
Supplement: Supplementary file 2 — Appendix S1: supplementary material for review [file EM-60-331-s002.docx]

<https://www.dropbox.com/sh/bhoi04ots44yyh7/AADUVzzkAIpAhFb8UfkrdXoua?dl=0>

The above is link to a video intended for EMM-18-0102, as supplied by author Julie Cox. This video was too large a file to upload into Manuscript Central; please click and view the video instead. It is supplementary material for review.
